# Inorganic Nanomaterials versus Polymer-Based Nanoparticles for Overcoming Neurodegeneration

**DOI:** 10.3390/nano12142337

**Published:** 2022-07-07

**Authors:** Simona Martano, Valeria De Matteis, Mariafrancesca Cascione, Rosaria Rinaldi

**Affiliations:** Department of Mathematics and Physics “Ennio De Giorgi”, University of Salento, Via Arnesano, 73100 Lecce, Italy; simona.martano@unisalento.it (S.M.); ross.rinaldi@unisalento.it (R.R.)

**Keywords:** neurodegeneration, neuroprotection, nanocarriers, blood–brain barrier, brain-targeting, drug-delivery, antioxidants

## Abstract

Neurodegenerative disorders (NDs) affect a great number of people worldwide and also have a significant socio-economic impact on the aging population. In this context, nanomedicine applied to neurological disorders provides several biotechnological strategies and nanoformulations that improve life expectancy and the quality of life of patients affected by brain disorders. However, available treatments are limited by the presence of the blood–brain barrier (BBB) and the blood–cerebrospinal fluid barrier (B–CSFB). In this regard, nanotechnological approaches could overcome these obstacles by updating various aspects (e.g., enhanced drug-delivery efficiency and bioavailability, BBB permeation and targeting the brain parenchyma, minimizing side effects). The aim of this review is to carefully explore the key elements of different neurological disorders and summarize the available nanomaterials applied for neurodegeneration therapy looking at several types of nanocarriers. Moreover, nutraceutical-loaded nanoparticles (NPs) and synthesized NPs using green approaches are also discussed underling the need to adopt eco-friendly procedures with a low environmental impact. The proven antioxidant properties related to several natural products provide an interesting starting point for developing efficient and green nanotools useful for neuroprotection.

## 1. Overview

### 1.1. Neurodegenerative Diseases (NDs): Properties and Conventional Treatments

**Figure 1:**
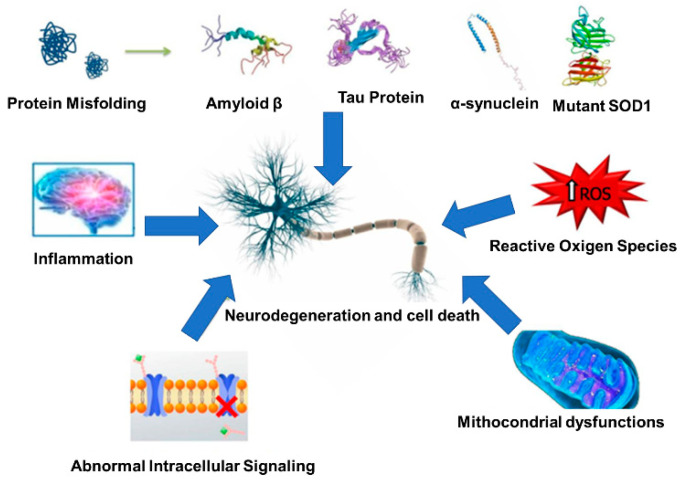
Processes that lead to neurodegeneration and cell death: protein misfolding (specifically related to Amyloid β, Tau protein, α-synuclein, and SOD1), increase in reactive oxygen species, mitochondrial dysfunction, inflammation, and aberrant intracellular signaling.

NDs are characterized by a progressive loss of neurons and glial cells and their networks in the brain and/or spinal cord. NDs can be generally classified according to different criteria: known etiology (i.e., genetic mutations or sporadic forms), clinical presentation, anatomical regions and cell types injured, altered processing, misfolding, and aggregation of the proteins involved in the pathogenesis of the disease [1]. Also, according to the involved proteins, NDs can be distinguished into tauopathies, α-synucleinopathies, TDP-43 proteinopathies, FUS/FET proteinopathies, prion diseases, trinucleotide repeat diseases, neuroserpinopathy, and ferritinopathyand cerebral amyloidosis [1]. Disorders such as Alzheimer’s (AD), Parkinson’s (PD), Huntington’s (HD), Multiple Sclerosis (MS), and Amyotrophic Lateral Sclerosis (ALS), reveal a series of clinical features related to different regions of the central nervous system (CNS). The typical outcomes are memory and cognitive disturbances (dementia), whereas others affect motor (ataxia), speech, and breathing capacities or a combination of these is also common. In detail, alterations in high-order brain function can also occur (i.e., affecting the hippocampus, limbic system, and neocortical areas). Among motor disorders, hyperkinetic, hypokinetic, cerebellar, and dysfunction of the upper and lower motor neurons (impacting the basal ganglia, thalamus, cerebellar cortex and nuclei, motor cortical areas, and lower motor neurons of the spinal cord) are also peculiar [1]. In MS and other neuro disorders, a shared cellular response is the glial scar formation. A glial scar consists of a severely damaged brain area surrounded by reactive glial cells [2]. Only a clearer comprehension of the mechanisms and etiology of each disease can promote the advancement of innovative and more effective treatments [3]. BBB breakdown is a common hallmark observed in many neurological diseases, allowing structural and functional changes in the microvasculature. In general, these alterations developed before neurodegeneration, persisting during the disease progression [4]. In this context, other factors contribute to neurodegeneration: protein misfolding, oxidative stress due to the generation of reactive oxygen species (ROS), mitochondrial dysfunction [5], and abnormal intracellular pathways (e.g., altered expression of drug transporters, aberrant neural signaling, and ion homeostasis) [6] (Figure 1).

Alzheimer’s disease (AD) is one of the most common progressive neurodegenerative disorders that leads to dementia among the elderly. The first clinical symptoms are usually described as short-term memory loss, cognitive impairment, behavioral disturbances, and physical impairment [7]. Typical pathological hallmarks can be identified in the extracellular deposition of amyloid plaques, consisting of aggregates of amyloid-beta (Aβ) peptides and intracellular storage of neurofibrillary tangles (NFTs), which are aggregates of hyperphosphorylated tau protein [8]. 

Currently, available treatments are based on acetylcholinesterase inhibitors (donepezil, rivastigmine, and galantamine), which act at the synaptic level [9] and are highly unstable in blood. Also, memantine is used for the most severe forms of the disease. However, in vitro and/or in vivo studies have reported the potential use of natural compounds as therapeutic agents such as sesamol, curcumin, huperzine A, phosphatidic acid, resveratrol, grape seed extract, or alpha-mangostin [10]. Previously induced cognitively impaired rats with intracerebroventricular (ICV) streptozotocin (STZ), were treated with sesamol solid lipid nanoparticles (SLNs). The study revealed much more efficacy compared to the group of rats treated with plain sesamol at a 16 mg/kg dose, which was almost analog to the conventional rivastigmine one. Hence, their behavioral and biochemical findings have shown an alternative to alleviate ICV-STZ-induced neuronal dysfunction and memory deficits by administering sesamol SLNs [11]. JA Loureiro and colleagues provide evidence that grape skin and grape seed extracts allow a pronounced inhibition of Aβ (1–42) aggregation, suggesting the synergistic effect of other polyphenols with resveratrol. They also developed and tested extracts encapsulating SLNs decorated with an antibody (OX26) that facilitates the BBB crossing [12].

The FDA-approved drugs for AD therapy cannot prevent the progression of the disease but can temporarily increase cognitive function, ameliorating cholinergic and glutamatergic neurotransmission [7]. Therefore, the need to discover and process new therapeutic strategies is a primary goal for overcoming the limitations of available drugs. Parkinson’s disease (PD) occurs in adulthood and is caused by dopamine loss triggering motor symptoms such as balance problems and tremors, mainly during rest. Classical parkinsonian symptoms are associated with the loss of dopaminergic neurons in the substantia nigra and with intracellular inclusions containing aggregates of α-synuclein (Lewy bodies) [10]. Heterogeneous symptomatology is currently treated by directly stimulating dopamine receptors or increasing dopamine concentrations [13]. Levodopa, the precursor to dopamine, remains the most powerful therapeutic compound that is useful for motor symptoms, but additional strategies have also been referred [14]. Other substances that have been tested in in vitro and/or in vivo studies for PD include *N*-3,4-bis(pivaloyloxy)-dopamine (BPD), glial cell line-derived neurotrophic factor (GDNF), basic fibroblast growth factor (bFGF), coenzyme Q10 (CoQ10), resveratrol, and apomorphine andropirinole [10].

A promising approach to brain-targeting treatments as an alternative to levodopa is to adopt BPD, which is more stable than dopamine. This compound revealed improved brain-targeting due to the presence of RVG29, a peptide from rabies virus glycoprotein useful as a brain-targeting ligand, and showed therapeutic effects in a mouse model of PD. The construct BPD-RVG29-lip was reported in addition to the therapeutic efficacy, and there was also no systemic toxicity after intravenous administration, in the completed in vivo and ex vivo distribution studies [15].

Yue and coworkers investigated the effects of ultrasound-triggered GDNF plasmid-gene-loaded PEGylated liposome-coupled microbubbles (PLs-GDNF-MBs) on behavioral deficits and neuron loss in a rat model of PD. Using such a noninvasive method for inducing a transient BBB disruption, GDNF can act on dopaminergic neuron survival. This construct combined with MRI-guided focused ultrasound could be an effective way of delivering the GDNF gene directly into the brain [16]. Further studies are required; however, the technique represents a potential strategy for PD patients, based on the reported ameliorated behavioral deficits and neuron loss in the tested animal model. Amyotrophic lateral sclerosis (ALS) belongs to the ND family and allows a rapid and progressive deterioration of both lower and upper motor neurons that project from the brain and spinal cord [10]. These disturbances affect the signaling process between the motor neuron and muscle, resulting in gradual weakness and muscle wasting. The multifactorial pathogenesis involves different potential mechanisms that contribute to the neurodegeneration [10].

Huntington’s disease (HD) has an autosomal dominant inheritance caused by an expanded CAG trinucleotide repeat in the HTT gene on chromosome 4, encoding for the protein huntingtin [10]. The mutant huntingtin (mHTT) protein is associated with protein aggregation and toxicity, resulting in neuronal dysfunction and death, not only due to its intrinsic toxicity but also because it disturbs various cellular processes [17]. The aim of the treatment is to suppress the chorea, using the synaptic vesicular amine transporter inhibitor, tetrabenazine, as well as counteract the mood-altering features of the disorder [10]. Nowadays, there is no approved therapy able to modify or delay the progression of HD, but some substances, for instance, rosmarinic acid (RA) or P42 peptide, have been assessed in in vitro and HD rat models with modest results [10].

Fachel et al. reported on previous experimental work conducted by the Bhatt group focusing on the neuroprotective role of RA in HD. They formulated RA-loaded SLNs and then assessed them in vivo in a 3-nitropropionic acid (3NP)-treated murine HD model, proving the ability of RA-loaded SLNs in the reduction of movement deficits and striatal oxidative stress [18]. In detail, these ameliorations are related to impairments in body weight, beam walk, and coordination.

P42 is a 23aa peptide of the HTT protein and plays a role in preventing the aggregation and reduction of motor performance, and neurodegeneration in HD. Its activity in R6/2 mice has been assessed both in the pre- and post-symptomatic phases of the disease exerting therapeutic potential and effectiveness, respectively [19]. This therapeutic peptide is effective even in the case of overt symptoms and also has a direct impact on neuronal plasticity and activity. Thus, not only does it prevent the aggregation of mutant HTT in the early stages of the disease, but it also promotes some physiological functions of normal HTT, possibly treating HD. Multiple Sclerosis (MS) is a demyelinating autoimmune disease for which there is no currently effective re-myelination therapy [20]. In this chronic inflammatory condition, the destruction of myelinated axons in the CNS occurs and the related hallmark is the formation of plaque in the demyelinated nerve cells. Available therapies are designed only to ameliorate the outcomes and quality of life of affected people. However, there are FDA-approved disease-modifying immune therapies, such as interferons IFNb 1a and 1b and natalizumab, which slow the disease progression [21].

### 1.2. Blood–Brain Barrier (BBB)

**Figure 2:**
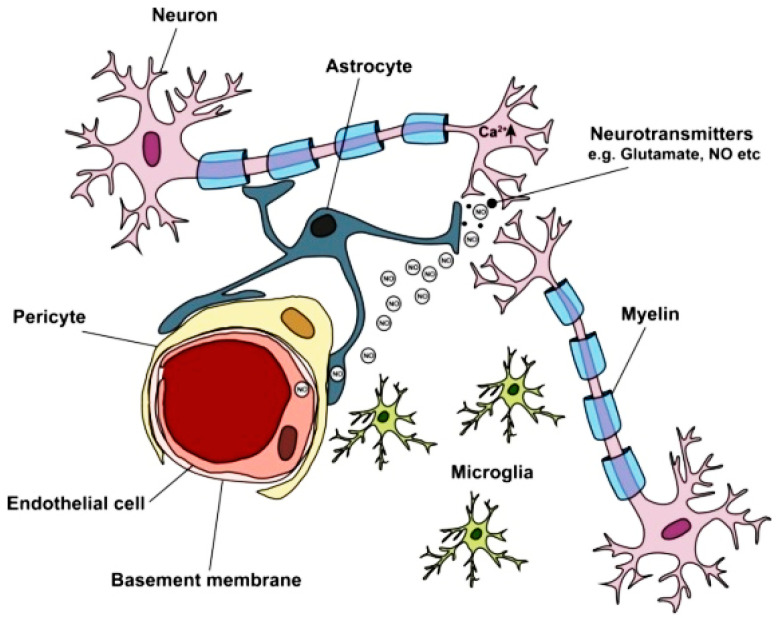
Diagram of the neurovascular unit (NVU). The neurovascular unit represents an interactive network of vascular cells (pericytes and endothelial cells), glia (astrocytes and microglia), and neurons. Adapted from [24].

The blood–brain barrier (BBB) acts as a complex blood–brain interface that separates the circulation system from the brain. Proper neuronal functions are ensured by its precise control of the CNS homeostasis, in addition to protection of the neural tissue against blood-borne agents, toxins, and pathogens. Therefore, changes in the properties of such a barrier affect the pathology and progression of different neurological diseases. This continuous anatomical structure consists of brain microvascular endothelial cells, which contact and stabilize pericytes, and astrocytes, which provide maintenance and repair support. In addition, tight junctions (TJs), neurons, and basal membranes are components of the BBB [7]. This barrier protects the CNS and strictly regulates the solute movement in the brain district [22]. A single layer of brain endothelial cells exerts a coating function on the microvessels located inside the brain parenchyma [23]. These endothelial cells are not fenestrated due to the presence of continuous intercellular TJs and adherens junctions that connect them; moreover, they also limit transcellular and paracellular transport due to their slow transcytosis process [10]. Cells having a vascular, and neural origin, as well as their complex relationships, contribute to the highly organized structure of the BBB, together constituting the recently named neurovascular unit (NVU) [24] (Figure 2).

**Figure 3:**
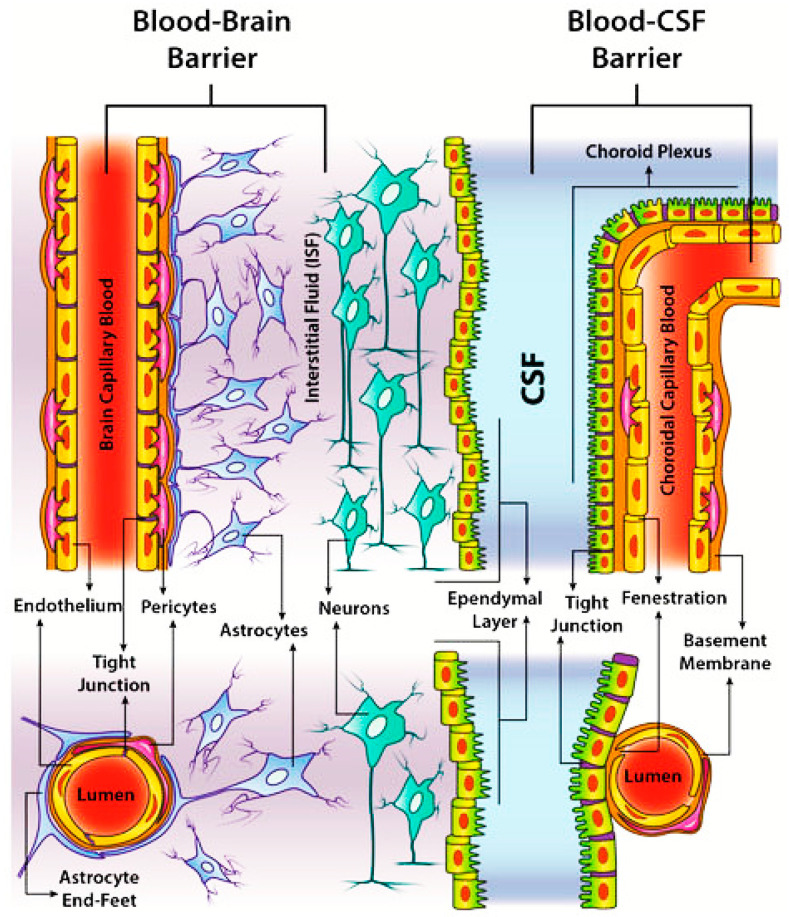
Representation of the main barriers for CNS drug delivery: blood–brain barrier (BBB) and blood–cerebrospinal fluid barrier (B-CSF). The cellular types are also shown. Reproduced from [29].

Considering the presence of this multifunctional barrier, the capability of such molecules to cross it strongly depends on drug-related and peripheral factors [10]. Generally, uncharged lipophilic molecules having <500 Da molecular weight (MW) and a partition coefficient in the range of 0.5–6.0 can overcome the BBB by a passive diffusion mechanism [7], whereas the lipophilic ones >600 Da MW and larger hydrophilic or lipid insoluble molecules are unable to cross the barrier [10]. The CNS is susceptible to abnormal processes that lead to the onset of neurodegenerative disorders (NDs), with related changes in the structure and functionalities of the microvasculature as well as dysfunctions of the BBB. From a mechanistic point of view, among the pathological processes involved in the onset and evolution of NDs, a pivotal role is attributed to genetic mutations and DNA damage, protein misfolding and aggregation, mitochondrial dysfunction, damage to the organelle membrane, apoptotic or autophagic cell death, and inflammatory responses [25]. Neuroinflammation is primarily triggered by microglia activation. Microglial cells are particularly responsive in the presence of imbalances in the brain homeostasis [26]. The characteristic late onset of most neurodegenerative disorders is highly associated with age, affecting the structure and functions of the BBB [22] and contributing to mitochondrial DNA mutations and redox dysregulation, thus leading to oxidative stress [25]. Many neurodegenerative conditions show a disruption to the integrity of the BBB; however, it is generally assumed that in AD patients, an increase in BBB permeability occurs in the presence of concurrent vascular dementia, probably as a consequence of alterations in the adherent junctions. A disrupted cerebrovascular architecture due to failures in the BBB allows for neuronal inflammation responses, with referred abnormalities in the lysosomal/autophagosomal and ubiquitin–proteasomal systems [27]. Cerebrovascular dysfunction and vascular pathology, in addition to BBB-damage-associated phenotypes, are particularly strongly associated with AD, PD, ALS, and MS [25]. However, in view of a specific treatment for NDs, the BBB is not the only structure that should be considered. In fact, the blood–cerebrospinal fluid barrier (B–CSFB) is the second critical barrier that should be analyzed with the aim of the systemic administration of drugs into the brain [28] (Figure 3). 

## 2. Application of Nanotechnology Tools for Neurodegeneration Therapy

### 2.1. General Aspects

Conventional drug-delivery systems provide suitable cellular structural repair and connection networks, crucial for the functional recovery of NDs but are considered palliative. Therapeutic drugs and biomolecules are strongly limited by the presence of the BBB, making them less efficacious for regular treatments [30]. The possibility of conjugating therapeutics from NPs, such as nucleic acids or drugs, has created new perspectives for target-specific nanomedicine. At the target site, the interactions established between the nanostructures and biological systems at the molecular level, allow physiological beneficial responses while minimizing adverse effects.

In terms of a treatment strategy, nanomedicine represents an efficient and promising tool for overcoming the limitations that challenge traditional medicine. In this respect, this approach has the potential to eradicate diseases linked to neuronal damage by helping patients worldwide to live a normal and healthy life. Available symptomatic treatment therapies, such as inhibitors of anti-cholinesterase, only slow down and manage the symptoms, rather than counteracting the causes and progression of the disease [31]. Moreover, other employed strategies are based on disease-modifying treatments, adopting, for instance, anti-inflammatory or antioxidant agents. For example, 75–200 mg/day of tetrabenazine alleviates involuntary movements (chorea) in PD patients; on the other hand, this compound acts as a vesicular monoamine transporter inhibitor (VMAT), thus leading to the appearance of neuropsychiatric-based symptoms as a side effect [31]. Furthermore, L-Dopa, a first-line treatment in PD, often causes several adverse effects and does not slow down the progression of the disease. Also, Donepezil, a cholinesterase inhibitor, is minimally effective in ameliorating cognition in AD [31].

In this regard, the development of novel therapeutics is urgently required, and nanomedicine can offer advanced therapies to overcome the current disadvantages of available traditional therapies, for instance, by bypassing non-specific targeting.

Considering this, the scientific community has been focused on the use of phytochemicals thanks to their minimal side effects. In fact, their intrinsic antioxidative, anticholinesterase, anti-inflammatory, and anti-amyloid properties make such chemicals promising therapeutic agents as plant-based drugs for nanomedicine [31].

The complex clinical translation in NDs is due to a multitude of factors: the absence of proper biomarkers, unclear molecular pathogeneses, well-timed diagnoses, the absence of disease models, and the heterogeneous outcomes of the disease [25]. Some NP-based treatments are currently in use, although none of them are applied for neurodegeneration. 

Nevertheless, NPs and their manipulations (i.e., to interact with serum proteins, to adapt their electrostatic interactions, hydrophobic domains, and adding peptides) require further studies in addition to toxicity and safety issues [32].

Also, mandatory approval following in vivo treatments, falls under agencies such as the FDA or the European Commission. They evaluate the toxicity, biocompatibility, and functionality features related to new kinds of therapies. Moreover, the preclinical and clinical stages prior to a drug’s approval are long processes, a parameter that should be considered in view of developing a timely treatment.

### 2.2. Nanomaterials As Active Therapeutic Agents

**Figure 4:**
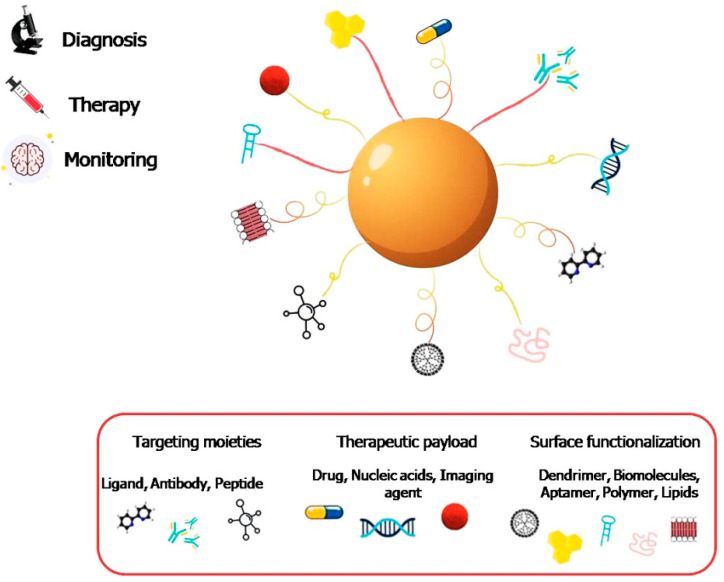
Design and application fields of multimodal theranostic nanoparticles. Targeting moieties, therapeutic payload, and surface decoration are exemplified in the picture, providing a series of useful tools to improve clinical outcomes.

In recent years, nanomaterials have emerged with broad applications in several biomedical fields such as drug delivery, biosensors, bioimaging, and neuro nanomedicine. Nanomaterials applied in the theranostic field can be specifically functionalized by adding different chemical constructs: targeting moieties (useful for selectively targeting cells and action sites), therapeutic agents (mainly for drug delivery), noninvasive diagnostic agents, and polymer coatings or matrixes that provide colloidal stability as well as functional groups for bioconjugation (Figure 4).

Nanomaterials are used due to their high surface area to volume ratio, associated with interesting physical, optical, and electrical properties. In this way, it is possible to apply these nanotools as diagnostic and therapeutic agents that can be adsorbed, dissolved, or covalently linked to the surface, obtaining NP–drug/imaging/targeting conjugates [33]. The CNS consists of different highly organized subtypes of neurons and glial cells, and the complexities associated with NDs influences the variability in terms of the diagnosis [34]. In this regard, nanomaterials as nanoparticles (NPs) represent advantageous tools considering their stability, biocompatibility, effective targeting, noninvasiveness, low toxicity, and the possibility to control the encapsulation or release of the specifically loaded elements confined in their core [35]. Significant advances were made to develop nanotherapeutic tools able to cross the BBB for the diagnosis and/or treatment of the NDs. The main promising strategies to enhance brain uptake and drug release take place through a series of mechanisms. Moreover, the enhancement of the transcytosis rate across the BBB occurs in the presence of a surface functionalization by linking, for instance, targeting peptides or cell-penetrating ligands [36]. In this regard, peptide NPs were introduced as newer tools with the potential to be used in the treatment of CNS diseases [37]. The surface functionalization of NPs allows the improvement of the BBB passage and fosters brain delivery, also targeting molecular mechanisms depending on the type of disease. In this way, cells, cellular compartments, and intracellular or extracellular molecules, such as Aβ-plaques in AD, could be targeted. In this context, specific receptors located on the BBB recognize the apolipoprotein E (Apo E), allowing its internalization into the brain. Then, functionalizing NPs with lipoproteins, such as the conjugation of Apo E to albumin NPs or liposomes, would enable them to be recognized and transferred by specific receptors located on the BBB [38]. Two main generic pathways could be exploited to overcome the BBB and thus facilitate drug delivery to the CNS by “crossing” or “bypassing” it [7]. The “crossing” pathway is related to all the endogenous routes through which molecules can overcome the BBB; among them, paracellular transport, carrier-mediated transport (CMT), receptor-mediated and adsorptive-mediated transcytosis (RMT, AMT), and cell-mediated transport.

On the other hand, the “bypassing” pathway refers to all the administration routes that do not require direct physical interaction with the BBB. Based on invasive techniques, the direct injection of nanomaterials into the CNS can occur through intracerebroventricular (ICV), intraparenchymal/intracerebral, or intrathecal administration routes [7] or by inducing a temporary disturbance of tight junctions or adopting intracerebral implants (i.e., catheters, microchips). Such an invasive approach is particularly useful for brain tumors. Moreover, a noninvasive, reversible, time-controlled opening of the BBB can be achieved through external stimuli (e.g., ultrasounds), in addition to using vasoactive compounds (e.g., bradykinin) or osmotic solutions [10].

The research goal is to achieve nanocarriers able to remain stable in the bloodstream, playing a protective role for the drug and promoting long-term drug release, essential in the nanomedicine field. The active targeting of specific pathological cells is now a challenge for pharmaceutical nanotechnology; nanoengineered particles as nanodrugs possess the ability to cross the BBB and decrease in the invasiveness [39]. The most studied noninvasive brain drug-delivery materials are liposomes, polymeric NPs, and solid-lipid NPs (SLNs) due to their specific characteristics of biocompatibility, stability, low antigenicity, and high biodegradability [40].

In combination with new developments in BBB investigations, various approaches have been exploited to facilitate therapeutics delivery to the CNS, which can be classified into invasive and noninvasive techniques. Chemical noninvasive approaches consist of specific modifications of the drug structure to improve its chemical and physical properties, such as membrane permeation or solubility [41]. Some of the most applied chemical modifications are regarding pro-drugs and chemical drug delivery systems. In the pro-drugs strategy, the inactive form of the drug can pass through the BBB without difficulty, becoming metabolized into the active form inside the brain [42], as in the case of L-Dopa-encapsulated NPs [43]. Thus, the activation of pro-drugs only requires a single biochemical reaction, whereas chemical drug-delivery systems generally need a cascade of bioactivation steps. Chemical drug-delivery systems allow entry to the brain through the usage of lipophilic moieties [44] that are susceptible to rapid metabolic processes such as oxidation. This leads to a charged and highly polar intermediate molecule that prevents the rediffusion of the drug (still in an inactive conjugate form) out of the BBB [44].

Powerful neurotherapeutic agents could be administered intranasally (IN) to bypass the BBB through the olfactory pathway, as reported with Rosmarinic-acid loaded SLNs coated with polysorbate 80 (PS80) [45]. This approach could be an appropriate and effective strategy [22], depending on the amount of the drug that can be distributed to the brain, since the nasal cavity only permits a limited dosing volume. In addition, the colloidal carriers (i.e., liposomes, SLNs) carry molecules directly to the brain region and they are proved to be very effective against several CNS disorders [41].

## 3. NPs Uptake through BBB

Nanotherapeutics can cross the BBB, reaching the brain through transport mechanisms that strongly depend on their physicochemical features. Additionally, functionalized NPs can pass through the BBB, entering endothelial cells by endocytosis and distributing pharmaceuticals in the diseased brain. Here, the main transport mechanisms are briefly discussed [46].

The transcellular lipophilic pathway allows the free diffusion through the BBB of small lipophilic molecules (<400–500 Da), such as O_2_, CO_2_, steroid hormones, and alcohol, whereas many essential nutrients, ions, and hormones (e.g., electrolytes, vitamins, glucose, amino acids) can cross the BBB through carrier-mediated transcytosis (CMT). CMT usually transports small molecules from the blood to the brain and the most popular CMT transporter is GLUT1, which is highly expressed in the brain capillary endothelial cells. However, none of these receptors/transporters are exclusive to the brain, so the use of antibodies or peptides may affect immunogenicity and stability.

Another pathway is receptor-mediated transcytosis (RMT), which provides the selective transport of larger endogenous molecules, which are required for normal brain function and involve specific receptors such as receptors for low-density lipoprotein (LDL), transferrin (Tf), and insulin. Macromolecules or ligand-binding nanocarriers can pass through the BBB due to the presence of specific receptors on the luminal side of the barrier: LDL receptors (LDLRs), Tf receptor 1 (TfR1), insulin receptor, lactoferrin (Lf) receptor, glutathione transporter, and scavenger receptors class B type I.

Both the CMT and RMT mechanisms are highly dependent on specific receptors or transporters. Alternatively, many peptides or proteins, such as cell-penetrating peptides (CPP) or cationic proteins, can be delivered to the brain through adsorptive-mediated transcytosis (AMT). CPPs are short amphipathic or cationic peptides characterized by a high BBB-crossing capacity without the need for a receptor. Such a transport route is based on electrostatic interactions between the positively charged substrates and the negatively charged plasma membrane surface. Stem cells and immune cells, such as macrophages and monocytes, can cross the unbroken BBB, which displays tumor tropism in animal models.

After the uptake process in the brain, drug delivery may be compromised by efflux pumps, which extrude drug molecules back into blood circulation. These pumps, such as adenosine triphosphate (ATP)-binding cassette transporters, multidrug-resistant protein (MRP), and P-glycoprotein (P-gp), represent the natural protective mechanism of the brain to avoid exposure to foreign molecules. 

To enhance the BBB penetration of nanosystems, it is possible to promote BBB opening for the local delivery of large pharmaceutical agents by applying a series of external stimuli inducing hyperthermia or mechanical forces, for example, focused ultrasounds (FUS), near-infrared (NIR) ultrashort pulsed laser, or chemical modulators.

## 4. Antioxidants for Neuroprotection

In ND management, the most common antioxidant molecules studied for their neuroprotective role are Vitamins A, E, and C. Vitamin A represents a class of fat-soluble chemical compounds known as retinoids that includes retinol, retinal, and retinoic acid [47]. Vitamin A is not directly involved in the cerebral antioxidative mechanisms; however, a protective role against amyloid fibrillation-associated cytotoxicity was demonstrated. The protection property emerged from the interaction with the Aβ 42 peptide, thus representing a potential strategy to counteract systemic amyloidosis [48] for Alzheimer’s and Parkinson’s treatments. Vitamin E or α-tocopherol can cross the BBB acting as a ROS scavenger in brain cells, which prevents lipid peroxidation [47]. Animal studies were performed on Sprague Dawley rats. The efficiency of vitamin E in neurodegeneration-induced chronic cerebral hypoperfusion was reported as a neuroprotective and antioxidant agent [49]. A vitamin-E-loaded resveratrol nano emulsion was formulated to target the brain, guaranteeing the synergistic effect of both compounds in the treatment of PD after IN administration. An enhanced CNS availability was reported, thereby reducing the systemic availability of resveratrol [50]. Vitamin C, also known as ascorbic acid, is involved in vital functions in the body. It is abundant in the brain where it takes part in physiological phenomena such as neuronal differentiation, maturation, survival, and neurotransmission modulation [47]. Several studies have shown that vitamin C deficiencies could be related to neurodegenerative disorders, including PD, AD, HD, and ALS [47]. In detail, a clinical study showed that the administration of vitamin C and/or E supplements resulted in a decreased risk of cognitive decline in people ≥65 years old [47]. Another investigation studied the neuroprotective effect of vitamin C in chronic restraint stress-induced rats, which improved synaptic activities and cognitive function. Moreover, findings from experiments performed on a *Drosophila* model with PD-like phenotypes, have shown that the administration of vitamin C at high doses leads to significant side effects; in addition, long-term treatment could prevent the degeneration of dopaminergic neurons [51]. ascorbate-conjugated nanocarriers were developed by Salmaso et al. [52] to selectively target the ascorbate transporter (SVCT2) expressed in the epithelial cells of the choroid plexus. This carrier acts to filter vitamin C into the CNS and, in some brain tumor cell lines, can be exploited as a potential target.

Carotenoids are a family of lipid-soluble pigmented compounds synthesized primarily in plants and algae but also by microorganisms, such as yeasts, fungi, *archea*, and *eubacteria*. 

Their chemical structure, with a long carbon chain of conjugated double carbon–carbon bonds, is highly reactive with free radicals, making them powerful antioxidant agents [47]. Numerous studies were assessed to evaluate the neuroprotective role of several compounds such as lycopene, astaxanthin, fucoxanthin, and crocin. Lycopene inhibits neuroinflammation by suppressing COX-2 and NF-κB and activating protein-1 and heme oxygenase-1. Similarly, astaxanthin inhibits lipopolysaccharide (LPS)-induced neuroinflammation, oxidant activity, and amyloid genesis in mice models, and also prevents hippocampal insulin resistance and AD complications in Wistar rats [47]. Crocin was administered for the therapy of AD and PD resulting in a potential treatment for neurodegeneration [53]. 

Phenolic compounds are the most widely available secondary metabolites in plants, characterized by phenol units providing antioxidative properties due to their free radicals’ scavenger activity and hydrogen atoms as well as electron donators, the chelating agents of metal cations [47].

They are known for their effects on mediating neuroinflammation and NDs by targeting the toll-like receptor (TLR) and could be used as pharmacophores in the development of therapeutic strategies for the treatment of neurological disorders [47].

These compounds are classified into flavonoids and non-flavonoids [54]; the first ones have a lipophilic property that allows their permeation through the BBB. Green materials such as fruits, vegetables, cocoa, dark chocolate, and beverages, such as red wine and tea [54], are rich in these compounds. 

Two kinds of flavonoids isolated from *Trigonella foenum* extracts, i.e., amurensin and cosmosiin, act against NaNO_2_-induced neurodegeneration in mouse brains, proving their role in inhibiting neurodegeneration in the hippocampus and cortex regions [47].

In addition, proanthocyanidin is proven to mitigate rotenone-induced oxidative stress in human neuroblastoma SH-SY5Y dopaminergic cells [47], a model for Parkinson’s disease.

On the other hand, tannins, coumarins, lignans, quinones, stilbens, and curcuminoids are defined as non-flavonoids [55]. Dietary polyphenols have been shown to reduce the oxidative stress involved in the onset and progression of neurodegeneration [47]. In this context, a research group investigated the effects of polyphenols, including gallic acid and ellagic acid at physiological concentrations, against H_2_O_2_-induced oxidative stress on human neuroblastoma SH-SY5Y cells [47]. The administration of these compounds prevented neuronal apoptosis, by reducing ROS levels, preventing caspase-3 activation, and increasing redox activity [56]. Another study reported the neuroprotective effect of gallic acid isolated from *Sanguisorbae* radix extracts against amyloid β protein-induced toxicity in rat cortical neuron cultures [57]. Furthermore, wine-derived phenolic compounds have exhibited neuroprotective effects on SH-SY5Y neuroblastoma cells by inhibiting caspase-3 activity and preventing reactive nitrogen species (RNS)-induced stress injury [58]. 

**Figure 5:**
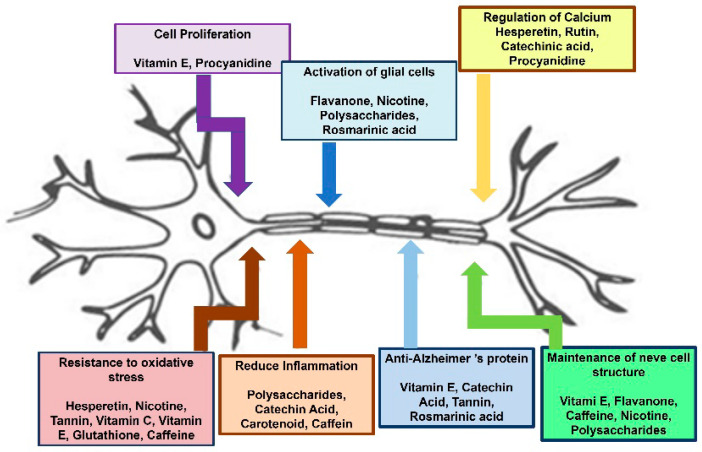
Protective method of plant-derived active substances on nerves. Active plant substances exhibit rich neuroprotective methods, which can be grouped into seven categories: promote the activation of glial cells, promote cell proliferation, regulation of Ca^2+^, maintain nerve cell structure, provide resistance to oxidative stress, reduce inflammation, and anti-Alzheimer’s protein. The active substances falling into each of these seven categories are provided in the respective boxes. All the active substances could resist oxidative stress, and some substances have similar protection patterns.

All these antioxidant compounds act through different mechanisms of action, as depicted in Figure 5.

## 5. Nutraceutical-Loaded NPs and Green NPs

In recent decades, nanotechnologies have been approaching the synthesis of NPs using green chemistry, which represents an alternative cost-effective and eco-friendly synthetic strategy. This approach replaces the toxic chemicals used as the reduction and stabilization agents of NPs with phytochemicals, which preserve biocompatibility and the bacteriostatic properties. Considering the enhanced brain targeting associated with drug delivery, several nanoformulations were implemented, overcoming the typical biological and chemical limitations that affect this kind of compound. Flavonoids are natural compounds derived from plants that have health-promoting effects; flavonoid-loaded NPs were designed and tested, confirming their antioxidant, anti-inflammatory, and neuroprotective properties [7]. In neurodegenerative models of AD, HD, PD, ALS, and MS, various flavonoids have been tested due to their therapeutic potential [8] for improving motor functions and producing neurotrophic factors, while decreasing oxidative stress, lipid peroxidation, and inflammation and preventing cognitive deficits [8]. So, flavanols could potentially be used as pharmacological agents for preventing neurodegeneration combined with a precise strategy to improve the passage through the BBB. Among these natural compounds, resveratrol is a free-radical scavenger and an acetylcholinesterase inhibitor derived from grape skin and seeds [7]. It was demonstrated that resveratrol is efficient in the attenuation of neuronal injuries and the modulation of neuronal signaling and glial pathways, as well as in the autophagy process [7]. Furthermore, resveratrol nanocarriers obtained by encapsulating or conjugating it in SLNs or nanocapsules, have shown improved solubility and a slower metabolism associated with their capability to efficiently target and inhibit the formation/aggregation of Aβ peptides [59]. Kanubaddi and colleagues reported improvements in the stability and solubility (dependent on the hydrophobic nature) of curcumin, a turmeric derivative belonging to the ginger family, which can bind to and dissolve Aβ aggregates when conjugated with AuNPs, nanogels, polymeric NPs, and nanoliposomes [59]. 

An innovative curcumin nano delivery system with high encapsulation efficiency, loading capacity, and better stability is represented by zein-hyaluronic acid NPs (ZH-NPs) [60]. Zein (Z) is the major storage protein in corn and has been found exclusively in the endosperm. This is an alcoholic soluble protein, generally used for fabricating NPs to deliver bioactive compounds [61]. However, Z-NPs have poor stability when suffering from acid, base, saline ion, and heat treatment. In order to improve their stability, some biopolymers were used as a coating for the surface of Z-NPs [61].

Moreover, the association between different types of nutraceuticals could improve their preventive or therapeutic effects; in fact, flavonoids such as curcumin and quercetagetin can be co-delivered through layer-by-layer composite ZH-NPs [62]. Another promising strategy is to combine ferulic acid with curcumin and phosphatidylserine that together promote a synergistic therapeutic effect ameliorating cognitive dysfunction in AD mice [63]. From the perspective of neuronal regeneration, neurotrophic factors are molecules that enhance the growth and survival potential of neurons. They help develop neurons and take part in several processes in the mature nervous system such as synaptic plasticity and the formation of long-term memories. Many of these factors could positively promote neuronal proliferation and neurite growth [7] and their properties have been tested when combined with NPs. Nerve growth factor (NGF) is probably the most studied and it was tested in vitro by using several nanoformulations. An effective targeted delivery was achieved by applying an external magnetic field to iron oxide NPs (IONPs) combined with NGF [64]. In experimental studies, superparamagnetic iron oxide (SPIO)AuNPs, activated by a low-intensity light-emitting diode source [65], promoted neurite outgrowth and neuronal differentiation, enhancing the complexity of the neuronal branching network in neural crest-derived PC12 cells. 

An acceleration of neuronal differentiation by adding quercetin to this system (NGF-SPIO-NPs) [66] was also demonstrated. Moreover, AuNPs loaded with 6-mercaptopurine, an anti-inflammatory drug, and functionalized with the neuron-targeting peptide RDP, increased the cellular uptake, as observed in the human neuroblastoma SH-SY5Y cell line, thereby supporting cell proliferation and neurite growth [67].

## 6. Nanocarriers for Brain Targeting

**Figure 6:**
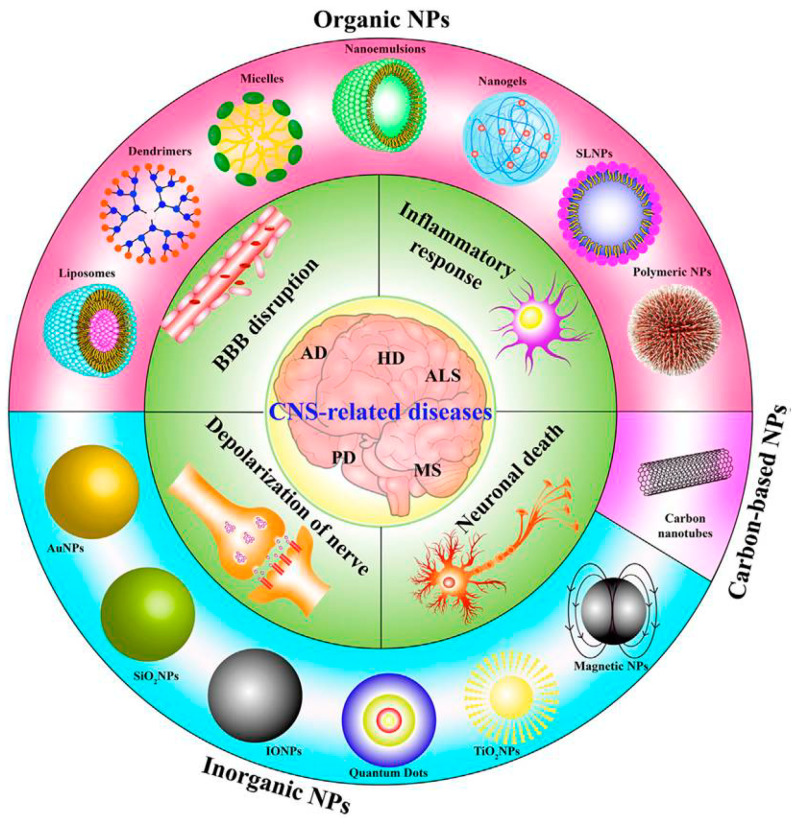
The potential therapeutic effects of NPs on the inflammatory response, neuronal death, depolarization of the nerve, and BBB disruption in CNS diseases including AD, PD, HD, ALS, and MS. Reproduced from [72].

Drug delivery for several CNS diseases is limited by the BBB. Precise and effective drug delivery to the brain represents a challenge in exploiting the appropriate physicochemical characteristics related to conventional neuropharmaceuticals, in terms of molecular size, lipid solubility, and surface charge [68]. NPs are susceptible to surface chemical functionalization; by tuning their interactions with endothelial cells at the brain level, it is possible to apply these nanotools in neuronanomedicine [69]. Therefore, loading therapeutic and imaging agents into specific nanostructures could overcome the typical limitations associated with conventional delivery methods across the BBB [70]. The intravenous (IV) administration route is the most suitable technique due to the nanocarriers’ ability to pass through the tissues and reach the CNS [71]. Currently, various types of nanocarriers each with a different chemical nature are available and have been adopted for neurodegenerative studies (Figure 6). Among the different nanostructures, organic NPs, inorganic NPs, and carbon-based NPs are the most widespread.

### 6.1. Inorganic NPs

Inorganic NPs, in particular metal, semiconductor, and metal oxide NPs, are characterized by unique intrinsic optical, electrical, and magnetic properties that have attracted the interest of the scientific community due to their potential biomedical application [73]. By tailoring specific parameters such as size, shape, composition, structure, and porosity, it is possible to improve their biological performance and functionalize their external surface through the ligands and polymers [73]. Silver NPs (AgNPs), iron oxide NPs (IONPs), and titanium dioxide NPs (TiO_2_NPs) are principally applied in bioimaging for disease diagnosis. However, several inorganic NPs, such as gold NPs (AuNPs) and silica NPs (SiO_2_ NPs), have been used as nanocarriers to reach the CNS [74]. Moreover, inorganic NPs are characterized by long-term enhanced permeability and the retention effect, which makes them a promising candidate for brain cancer treatment [73].

The advantages of these NPs for application in the field of medicine are that they are safe, hydrophilic, biocompatible, and highly stable under physiological conditions [7]. Metal NPs, such as AuNPs and AgNPs, show peculiar intrinsic optical properties known as Localized Surface Plasmon Resonance (LSPR) properties, whereas IONPs are characterized by unique magnetic properties. SiO_2_NPs and TiO_2_ NPs are inorganic ceramic NPs and guarantee higher thermal and chemical stability than polymeric ones [7]. IONPs, and Super Paramagnetic Iron Oxide NPs (SPIONs), can act as promising theranostic nanocarriers. In this regard, using magnetic resonance imaging (MRI), the inorganic core becomes detectable, serving as a contrast agent [7].

#### 6.1.1. Cerium Oxide Nanoparticles (CeO_2_NPs)

Cerium oxide (CeO_2_) NPs exhibited antioxidant properties leading to the degradation of the amyloid-β [75], a peptide whose accumulation in the brain is involved in the pathogenesis of Alzheimer’s disease. The CeO_2_NPs are well-tolerated NPs in in vitro and in vivo models, making them suitable for application in neuroprotection and regeneration [76]. An autocatalytic property is conferred due to the presence of a reversible Ce^3+^/Ce^4+^ redox system at their surface, responsible for their antioxidant role [70].

Recently, several green synthesis methods were developed for CeO_2_NPs based on natural and organic matrices as stabilizing agents to prepare biocompatible CeO_2_NPs. In this way, the green NPs could be potentially applied to nanomedicine. The plant-mediated synthetic routes of CeO_2_NPs are available using different plants such as *Gloriosa superba*, *Acalypha indica*, and even *Aloe vera* plant leaf extract [77]. The chemical compounds in plant extracts act as stabilizing and capping agents in the CeO_2_NPs’ synthetic process. This easy and cost-effective technique provides spherical-shaped NPs characterized by reduced cytotoxicity. Moreover, the biosynthesis of NPs using yeast and fungi has also been noted [77].

Thovhogi and coworkers synthesized spherical CeO_2_NPs from *Hibiscus sabdariffa* flower extract and their physicochemical properties were analyzed [78]. CeO_2_NPs were also obtained using fresh egg whites [79] as a bio-degradable eco-friendly capping and stabilizing agent, enriched by numerous amino acids and proteins such as ovalbumin. The interaction between the egg whites and the water and its ability to bind to metal ions makes the egg whites suitable for application as a shape-controlling stabilizing agent [79]. However, in vitro and in vivo-based research for brain targeting and delivery is required for the assessment of the thus-produced CeO_2_NPs using this last bio-directed method.

#### 6.1.2. Selenium NPs (SeNPs)

Selenium is an essential constituent element of key antioxidant enzymes. In mammals, there are 25 selenoproteins based on an active site with selenium. 

SeNPs are characterized by low toxicity and can induce a selective cytotoxic effect even in small amounts. Their peculiar polyvalent surface allows them to interact with various positively and negatively charged groups (NH, C=O, COO–, C–N, etc.), revealing a high adsorption capacity. Nevertheless, some toxic effects have been identified from SeNPs, which may challenge their potential use or require many clinical research studies. In fact, SeNPs exhibit pro-oxidant properties besides the ability to disrupt the cell membrane. It has been shown on Zebra fish embryos that 5–10 μg of SeNPs does not cause any harmful effects, whereas by increasing their concentration to 20–25 μg, several abnormalities occur (i.e., decreased heart rate and tail malfunction) [80]. The dose-dependent toxicity of SeNPs was also reported in *Daphnia magna* and the marine bacterium *Vibrio fischeri*. Following in vivo studies, it was proven that high doses of SeNPs allow for the abnormal storage of Se in organs such as the kidneys and liver, damaging them due to the promoted oxidative stress. Also, the size feature of SeNPs affects their antioxidant properties and their functionalization with other active substances. This is clearly shown in in vitro studies, observing a correlation between the small size (5–15 nm) and the enhanced free radical scavenging activity [80]. Several green starting materials have been used for the biosynthesis of SeNPs, for example, the leaf extract of *Aloe vera* and *Prunus amygdalus*, as well as other extracts of *Vitis vinifera*, *Allium sativum*, *Dillenia indica*, *Roselle* plant, *Cinnamomum zeylanicum* bark, fresh citrus, and lemons.

Many studies on neurodegeneration have revealed the ability of SeNPs to bind to Aβ, affecting their charges or reactivity, metal ions, and outer ligands [81,82].

In particular, SeNPs loaded with resveratrol (Res) improved the antioxidant and anti-aggregatory properties of such a natural compound, as demonstrated on PC12 cells, which specifically interacted with the surface of Aβ 42. As a result, the disrupted cell membranes lead to cell death. In general, Res@SeNP was found to be more effective than Res alone, which cannot inhibit PC12 cell apoptosis [80].

Chondroitin sulfate is a sulfated glycosaminoglycan involved in processes such as neurogenesis, axonal growth, synaptic plasticity, and neuro regeneration after injuries to the nervous system. ChS-loaded SeNPs (ChS@SeNPs) have been synthesized and tested in SH-SY5Y cells (human neuroblastoma), successfully protecting them from Aβ (1–42)-induced cytotoxicity and reducing the levels of ROS and malondialdehyde (MDA) and the hyperphosphorylation of tau (Ser396/Ser404) [80].

MPTP (1-methyl-4-phenyl-1,2,3,6-tetrahydropyridine) is a neurotoxin that is useful as a model for PD studies due to its peculiar role in the promotion of the degeneration of dopamine neurons and neurobehavioral disorders. The neuroprotective effects of produced glycine SeNPs were assessed in two animal model groups with or without MPTP. The potential therapeutic role of glycine SeNPs in PD was hypothesized thanks to the protective effect on oxidative stress of neurons through the regulation of key enzymes, such as Superoxide dismutase (SOD) and Glutathione peroxidase (GSH-PX), in addition to decreasing MDA levels [80].

#### 6.1.3. Gold Nanoparticles (AuNPs)

Gold nanoparticles (AuNPs) play an important role in different fields such as pharmacology, sensing, and bio-imaging. Although they are widely considered to be safe and are characterized by low phototoxicity, AuNPs still induce toxicity and their removal from the blood occurs through the hepatobiliary route [83]. The suppression of the pro-inflammatory responses in NDs was related to AuNPs tested on a microglial cell line. The induced polarization toward the M2 phenotype is considered beneficial for CNS repair and regeneration [84]. Starting from *Nigella arvensis* leaf extract, AuNPs with a size range of 3–37 nm were obtained through a one-step green method. Their antibacterial and antioxidant properties, as well as their cytotoxicity and catalytic activities, were assessed [85]. Also, using *Hypericum hookerianum* plant extract, AuNPs were produced and then tested in haloperidol (1 mg kg^−1^; intraperitoneally)-induced Swiss albino mice. A pronounced antiparkinson-like effect was observed due to the abundance of neuroprotective flavonoids in the extract [86]. Other researchers, such as Xue et al., synthesized AuNPs from the root extract of *Paeonia moutan* (PM-AuNPs), a woody tree that is used in traditional Chinese medicine due to its beneficial health properties for several disturbances. This extract consists of phytochemicals such as paeonol, paeoniflorin, oxpaeoniflorin, gallic acid, and others. The PM-AuNPs were tested both in vitro, in the murine microglial BV2 cells, and in vivo in Parkinson-induced C57BL/6 mice [87]. The results of the in vivo experiments authentically confirmed that these green NPs alleviate neuroinflammation and improve motor coordination in the tested animal model [87]. Microglial cells are resident macrophages, whereas BV2 cells derive from raf-/myc-immortalized murine neonatal microglia and are considered the best in vitro model used to evaluate the effectiveness of neurodrugs [88].

Moreover, biostable and bioactive AuNPs were obtained with procyanidin fractions from *Leucosidea sericea*, a frost-resistant evergreen tree, revealing an interesting antioxidant activity [89]. The potential beneficial effects of AuNPs synthesized from *Cinnamomum verum* on PD rat models were explored, exhibiting a decrease in induced oxidative stress and motor abnormalities, while activating inflammatory cytokines and TLR/NF-κB signaling [90].

Starting from *Ephedra Sinica* (ES) Stapf extract, AuNPs were obtained and then tested. A decrease in the levels of both pro-neuroinflammatory cytokines and mediators was reported, thus resulting in the amelioration of neurodegenerative disorders. Further, ES-AuNPs exhibited anti-neuroinflammatory properties by decreasing ROS levels in microglia when tested in mouse primary microglia and immortal BV-2 mouse microglial cells [91].

Ali T. et al. [92] investigated the effects of anthocyanin-loaded PEG-AuNPs in an Aβ (1–42) mouse model of AD. In the reported findings, both the anthocyanin-loaded PEG-AuNPs and anthocyanins treatment (12 μg/g/day for 14 days) ameliorated memory impairments in the Aβ (1–42)-injected mice [92]. However, the NP-based tool was more effective than free anthocyanins, resulting in a protective role in pre- and post-synaptic proteins from the Aβ (1–42)-induced synaptic dysfunction [92]. Then, a novel therapeutic approach was assessed to prevent age associated NDs by combining dietary polyphenolic compounds with AuNPs. 

Further studies conducted on nanomaterial-based treatments for NDs have demonstrated that AuNPs were able to inhibit amyloid fibril formation by reducing the α-lactalbumin protein in a molten globule state [40]. This protein is currently used as a sample for studying amyloid formation. The reported protective effect can occur due to the increase in protein adsorption to the AuNPs’ surface, preventing their structural changes. By binding AuNPs to the amyloid fibrils’ monomer, the aggregation process and core elongation, are inhibited, thus representing a useful tool for the prevention of amyloidogenesis and the treatment of related disorders [93].

Adopting a protein engineering approach, AuNPs conjugated to the specifically modified β-sheet breaker peptide, CLPFFD, could destroy the Aβ toxic aggregates. To modify the drug delivery ability of the AuNP-CLPFFD conjugate, the peptide sequence of THRPPMWSPVWP (identified using the nomenclature of the inserted amino acid residues) was introduced. The interaction between this amino acid residue sequence and the transferrin receptor located in the microvascular endothelial cells of the BBB is responsible for the increased permeability of the conjugate in the brain district [94]. AuNPs also revealed inhibitory effects on the fibrillogenic process of insulin fibrils and disrupted insulin amyloid fibrillation, further preventing shorter and more compact fibril types. Moreover, AuNPs can improve the acquisition and retention of spatial learning and memory in Aβ-treated rats; they are also associated with neural survival by increasing the expression of brain-derived neurotrophic factor (BDNF), cAMP response element-binding protein (CREB), and stromal interaction molecules (STIM) 1 and 2 [95]. Electromagnetic AuNPs in the presence of a specific electromagnetic field (EMF) stimulation, facilitated an efficient direct lineage reprogramming to induce dopamine neurons in an efficient and noninvasive way to ameliorate typical outcomes in PD models [96]. 

A ‘molecular surgery’ approach was found to regulate the progression of AD without damaging healthy brain cells through the destruction of β-amyloid fibrils and plaques [40]. The chemical procedure required to conjugate AuNPs to a β-amyloid fibril aggregate involves incubating the resulting mixture for seven days and then exposing it to 0.1 W and 12 GHz microwave fields at different growth stages for eight hours. The energy levels of the fields were found to be six times smaller than those of conventional cell phones and thus were not able to harm healthy cells. Subsequently, the fibrils that dissolved after the irradiation remained in this condition for at least one week, suggesting the efficiency of the treatment in breaking up the fibrils as well as the lower re-aggregation tendency of the proteins [97]. A similar approach could be extended for other NDs characterized by a protein aggregation phenomenon including Parkinson’s disease [97]. The approach is similar to a reported experimental method based on metal NPs to label and destroy cancer cells [98].

#### 6.1.4. Silver Nanoparticles (AgNPs)

Silver nanoparticles (AgNPs) showed tunable physical and chemical properties, which are widely applied in the biomedical field; among their numerous characteristics, the antibacterial and plasmonic features are the most important [99]. Biosynthesized AgNPs using green routes have been successfully made by using non-toxic solvents, such as water, and natural reducing agents such as glucose [100], dimethyl sulfoxide (DMSO) [101], gelatin and sugar, tea and coffee, and plant extracts [102]. These elements are able to replace some hazardous compounds often used in the conventional synthetic approach such as sodium borohydride and formaldehyde.

Some types of flavonols, such as quercetin, which were used in the synthesis of AuNPs and AgNPs have applications for the brain [103]. Several green routes can be applied to obtain AgNPs starting with different plants. Fresh aerial parts of *Lampranthus coccineus* and *Malephora lutea* were used for the biosynthesis of AgNPs. Their antioxidant properties and acetylcholinesterase inhibitor role were demonstrated with a comparable degree to rivastigmine. Moreover, in vitro assay evidence assumes the potential application of these biosynthesized AgNPs for AD therapy due to their anti-Alzheimer potential [104]. 

Other studies have demonstrated protocols useful for obtaining green, stable, well-distributed, crystalline, and spherical AgNPs (size 14–20 nm) through the bio-reduction of silver nitrate (AgNO_3_) using the leaf extract of a fast-growing tropical tree, *Melia azedarach* [105]. A comparative analysis conducted via DPPH and ABST assays confirmed the increased antioxidant activity of MA-AgNPs compared to the MA extract [105]. A sustainable eco-friendly approach was adopted to synthesize spherical-shaped AgNPs with an average size of ~73 nm starting with *E. suberosa* leaf extract. The achieved results strongly suggested the usefulness of AgNPs as natural ROS scavengers to preserve health and counteract degenerative diseases [106].

Green AgNPs synthesized from *Pulicaria undulata* L. were used in different concentrations to assess their effect on the aggregation of α-lactalbumin (α-LA) and the chaperone role of α_s_-casein. The results suggested that AgNPs prevented the aggregation of α-LA, which is used to study protein fibrils formation, in a dose-dependent manner. At the same time, they have no effect on the chaperone ability of α_s_-casein [107]. These results are useful from the perspective of the treatment of amyloidosis disorders.

It is known that AgNPs are able to reach the brain through the bloodstream after their in vivo administration due to their peculiar properties and also depending on their size. In particular, the NP distribution was evaluated at the BBB level in an in vitro model of rat brain microvessel vascular endothelial cells (BMVECs) cultured in a medium containing 100 μg/mL of either AgNPs or AgMPs (microparticles) [108]. After 4 h of exposition, only the AgNPs were able to overcome the BBB and accumulate inside the BMVECs [108].

The potential neuroprotective role of AgNPs with a size range of 1–100 nm, was reported in evaluating their role in the regulation of the gene and protein expressions of the Aβ depositions [108]. Furthermore, by setting up NP-labeled aptamers linked to PrP antibody (an anti-prion protein), it is possible to develop an interesting tool in the diagnosis of prion diseases, in which an abnormal folding of the prion proteins was found. In this regard, the AgNP–aptamer conjugates have permitted anti-prion protein detection [109].

Although several beneficial effects have been reported using AgNPs, it is important to offer a comprehensive view considering their potential side effects. In neural cells treated with AgNPs, the gene expression of amyloid precursor protein (APP) was induced [40]. In addition, the activation of the two main factors involved in the suppression of AD progression was reduced in neural cells together with their protein levels. The targeted factors were neprilysin, the major brain Aβ-degrading enzyme, and the low-density lipoprotein receptor, which enhances Aβ uptake and degradation at the brain level [40]. 

Also, systemic side effects have been investigated; Gliga et al. explored the size-dependent cytotoxicity of AgNPs in human lung cells, reporting the cytotoxic role of small AgNPs (10 nm) in human lung cells. In detail, the released ion fraction in the cell medium did not induce any cytotoxicity, suggesting that the rate of intracellular Ag release was responsible for the toxicity [110].

In another work, the particle size of chitosan nanoparticles (CS-NPs) was recognized as responsible for the cytotoxicity effect of CS-NPs on mouse hematopoietic stem cells (HSCs). An indirect influence was also determined by the chitosan concentration and molecular weight [111]. 

#### 6.1.5. Magnetic Nanoparticles (MNPs)

The superparamagnetic iron oxide core of magnetic nanoparticles (MNPs) allows the association of the imaging capability with the targeting property in the presence of a magnetic field. In particular, by coating the MNPs with biocompatible polymers encapsulating specific drugs, targeted drug delivery was assured, measuring at the same time the targeting efficacy through MRI [112]. By employing biocompatible materials, MNPs with improved stability, reduced toxicity, and immunogenicity, can be synthesized. These nanomaterials are characterized by an intrinsic tendency to agglomerate due to their high surface energy [113], although this could be avoided through electrostatic/steric repulsion. MNPs with a hydrodynamic radius <100 nm are not immediately recognized by mononuclear phagocytes [113]. Therefore, their long blood half-life and their ability to be a vehicle for the CNS, represent suitable features for potential CNS imaging and drug delivery associated with an applied magnetic field [113].

Experimental evidence showed that by applying magnetic therapy based on MNPs, the prolonged survival of glioma-bearing rats was demonstrated. In addition, an enhancement of the brain concentration of paclitaxel (a common antitumoral drug) occurred [114]. A similar in vivo study reported that magnetic targeting prolonged the retention of MNPs within gliomas, which adopted magnetic targeting. There was a fivefold increase in the accumulation of NPs within the targeted tumors compared to the non-targeted tumors [112].

Qiao et al. [115] demonstrated that the modification of PEG-coated MNPs with lactoferrin induced enhanced permeability across the BBB by exploiting receptor-mediated endocytosis via an interaction with the lactoferrin receptor on brain endothelial cells. 

The interaction between IONPs with astrocytes has been extensively investigated. In particular, SPIONs are typically based on Fe_3_O_4_ and/or Fe_2_O_3_ and they are widely used for biomedical applications, including magnetic resonance imaging (MRI), magnetic particle imaging (MPI), magnetic fluid hyperthermia (MFH), and targeted drug and gene delivery [116]. The term “superparamagnetic” means that at specific temperatures in the absence of an external magnetic field, the magnetization of these NPs appears to be zero on average (i.e., in the superparamagnetic state). So, by applying an external magnetic field it is possible to induce the magnetization of the NPs due to their very high magnetic susceptibility [113].

They can be functionalized with biocompatible materials, drugs, proteins, or plasmids. When an external magnetic field was applied, SPION coated with reversibly bound ligands could be used to target specific action sites [40].

However, SPIONs exhibit unfavorable pharmacokinetic behavior leading to liver and spleen accumulation due to the opsonization and scavenging by the mononuclear phagocyte system. For this reason, ultra-small SPIONs (USPIONs) have been developed for drug-delivery applications [73]. The exploitation of external stimuli, including through near-infrared-region (NIR) radiation and magnetic fields, allows the brain uptake of these magnetic particles, which improves tissue imaging and could also enhance on-demand drug release across the BBB [73]. The application of a low radiofrequency field to the IONPs allows the generation of thermal energy that is responsible for the transient and local open-up in the BBB [117]. 

By conjugating SPIONs with various compounds, it is possible to facilitate their active transport to the brain due to the absence of passive transport. In this regard, quercetin-conjugated dextran-coated SPIONs (QT-Fe_3_O_4_ NPs) were prepared by a chemical nanoprecipitation method. The advantage of using SPIONs is their ability to enhance the bioavailability of quercetin. Wistar male rats, used as animal models, were orally fed with QT and QT-SPION at 50 and 100 mg/kg daily doses for 7 days [118]. The results of the study revealed a higher concentration in the plasma and brain of the rats fed with QT-SPION compared to free QT. After the QT-SPION administration, a tenfold higher bioavailability of quercetin in the brain was detected than free QT, thus confirming that SPIONs act as a targeted drug delivery system. This is useful in light of a potential treatment for neurodegenerative disorders [118]. Many symptoms of human diseases can be reproduced in rats; Wistar rats are a purebred species widely used in medical testing due to their biological, genetic, and behavioral features that are closely related to those of humans [118].

Liu et al. reported a multifunctional SPION conjugated with Aβ oligomer-specific scFv antibody W20 and class A scavenger receptor activator XD4 (W20/XD4-SPIONs). W20/XD4-SPIONs retained the anti-Aβ properties by inhibiting Aβ aggregation, attenuating Aβ oligomer-induced cytotoxicity, and increasing microglial phagocytosis of Aβ [119]. In APP/PS1 mice, W20/XD4-SPIONs significantly restored cognitive deficits and alleviated neuropathology in AD mice [119]. Then, a promising agent for early-stage AD diagnosis and intervention was presented.

In another study, PVP-SPIONs were bio-conjugated with 1,2-Dimyristoyl-sn-glycero-3-phosphocholine (DMPC) [120]. Then, PVP-SPIONs and DMPC/PVP-SPIONs were co-incubated with rat adrenal pheochromocytoma (PC-12) cells to evaluate the effect of this phospholipid on the biodistribution of the SPIONs. Further, PVP-SPIONs and DMPC/PVP-SPIONs were implanted into the substantia nigra of Sprague–Dawley (SD) rats by stereotaxic injection, and the brain tissues were explanted at two time points after injection, i.e., at 24 h and 7 days after injection [120]. The lipophilic nature of these NPs allowed their attachment to bio-membranes in the brain. Also, a faster brain delivery of DMPC/PVP-SPIONs was reported compared to those without DMPC. Good biocompatibility and biodegradability properties resulted from the in vitro assays, with a related ability to activate neuron membrane channels [120].

### 6.2. Organic Nanoparticles

Polymeric NPs are defined particles with a size ranging from 10 to 1000 nm, which is relatively easy to achieve [73]. These NPs are largely applied based on the selection of specific biocompatible and biodegradable polymers as well as those of a synthetic origin. Among synthetic polymers, polylactic acid, polyglycolic acid, polylactide-co-polyglycolic acid, poly(ε-caprolactone), and polymethyl methacrylate are mostly used [73], whereas chitosan, alginate, gelatin, and albumin [121] belong to the class of natural polymers. Specific parameters, such as the structure of the polymer and the entrapping method, affect the pharmacokinetic behavior of the encapsulated agents [122]. Their relevant use is related to drug delivery as drug carriers thanks to their higher drug-loading and drug-protection skills [123]. Specifically for AD and PD, polymeric NPs are employed due to their ability to open TJs, crossing the BBB and targeting the mutagenic proteins. In addition, these stable particles can also be manipulated to avoid recognition by macrophages of the reticuloendothelial system (RES) [124]. Several drugs, such as dalargin, loperamide, and doxorubicin, were driven to the CNS using these polymeric systems. Generally, drugs used to treat NDs are characterized by their small molecular weight (150–500 Da), and highly lipophilic properties, which promote penetration through the BBB [123]. In addition, NP-based drug-delivery systems require the checking of several issues for an accurate design, for example, the nature of the drug within biological systems, the enzymatic degradation in the peripheral circulation, the removal of NPs by the efflux systems, possible cellular retention, and the clearance rate of drugs at the brain level [123]. A polymeric system developed for application in biomedical or environmental fields should be completely free from additives or reactants, such as surfactants or traces of organic solvents, which are hazardous to the environment as well as to physiological systems [125]. In this view, it is preferable to adopt synthesis techniques such as RESS (rapid expansion of a supercritical solution) or RESOLV (rapid expansion of a supercritical solution into a liquid solvent) due to the absence of any surfactant or organic solvent in the applied protocols [125]. Polymeric NPs used as nanocarriers are made of a matrix architecture that is generally represented by nanocapsules and nanospheres.

#### 6.2.1. Poly-Butylcyanoacrylate (PBCA) NPs

Poly-butylcyanoacrylate (PBCA) NPs coated with polysorbate 80 (PS80) and conjugated with Enkephalins (neuropeptide) and Doxorubicin (drug), were taken up by brain capillary endothelial cells via receptor-mediated endocytosis. The key role of the polysorbate coating is to adsorb the apolipoproteins B and E from the blood on the NPs’ surface [37].

In particular, degradable NPs have become the main type of neurodegenerative drug carriers due to their low toxicity, tunable degradation rates, and high drug-loading capacity, as well as their ability to pass through the BBB and target the CNS [126]. *N*-butylcyanoacrylate NPs and PBCA NPs encapsulating quinoline derivatives have been developed. Among these derivatives, Clioquinol (CQ) is a Cu^2+^/Zn^2+^ chelator known to solubilize the β-amyloid plaques in vitro and inhibit the β-amyloid accumulation in AD transgenic mice in vivo [123]. Emphasis was placed on the prospects of CQ NPs as vectors for the in vivo brain imaging of β-amyloid senile plaques due to the capability of these nanocarriers to freely cross the BBB without additional intermediates. A few studies have reported the effective crossing of the BBB of L-DOPA-encapsulated NPs and the subsequent decrease in PD basic symptoms, as suggested in Ref. [43], and in PBCA NPs conjugated with nerve growth factor (NGF) [127]. Assuming that metal ions are known to bind to Aβ and alter its solubility, metal chelators such as CQ, ethylenediaminetetraacetic acid (EDTA), and desferrioxamine, have shown improvements in clinical studies conducted on AD patients [128]. In particular, in a two-year single-blind study, the progression of dementia was investigated by administering desferrioxamine (125 mg intramuscularly twice daily, 5 days per week, for 24 months) and compared to an oral placebo (lecithin) and an untreated group. A relevant decline in basic skills was found to be associated with the chelating treatments [128]. However, their clinical usefulness was limited due to their toxicity and lower bioavailability caused by low penetration through the BBB. It is possible to overcome these limitations by using NPs, in particular, Nano-N2PY, an NP–chelator conjugate, was synthesized starting with polystyrene NPs. This conjugate has exerted a protective role on human cortical neurons against the toxicity of the Aβ oxidation [129]. D-penicillamine, a metal chelator, was approved by the US FDA to treat Wilson’s disease, as well as rheumatoid arthritis, despite its high hydrophilic nature and rapid removal from the blood [130]. Also, in this case, this compound was covalently conjugated with NPs and transported into the brain to evaluate its effectiveness in resolubilizing Aβ–copper aggregates in in vitro assays. In the presence of the reducing agent dithiothreitol, the chelator release occurred, and the Aß–copper aggregates were resolubilized [130]; however, further studies in animal models are required. 

#### 6.2.2. Poly (Lactic-Co-Glycolic Acid) (PLGA NPs)

PLGA is a copolymer of poly lactic acid (PLA) and poly glycolic acid (PGA). Generally, surface-modified PLGA NPs with polysorbate 80 (PS-80) and poloxamer 188 have shown improved CNS penetration [131]. PEG-PLGA NPs, when modified at the surface level with lactoferrin, showed the optimal delivery of rotigotine, a dopamine agonist, through intranasal administration to the brain, for the treatment of PD [132]. Through cellular uptake experiments, higher storage of lactoferrin NPs compared to naked NPs was reported in human neuroblastoma (SH-SY5Y) and human normal bronchial epithelial (16HBE) cell lines [37]; however, free rotigotine was reported to be cytotoxic. This study highlighted the great potential of lactoferrin NPs as a carrier of rotigotine from the nose to the brain for the treatment of PD patients [37]. Also, coumarin-6-loaded lactoferrin (Lf)-conjugated PEG-PLGA NPs reported a neuroprotective role for the same neurological disorder [133]. After injection of 60 mg/kg Lf-NPs or naked NPs in mouse caudal veins, the brain coronal section showed higher storage of Lf-NPs compared to free NPs in the cortex, substantia nigra, and striatum region [133].

In experimental studies, BMPs-9 (Bone morphogenetic proteins) led to a reduction in senile plaques, while enhancing cholinergic differentiation and maintenance. Also, growth factors, such as NGF (nerve) and IGF (insulin), are involved in the same process, and their role in tau hyperphosphorylation has also been described [130]. In this regard, PBCA NPs containing NGF were prepared and further coated with PS-80, achieving relevant memory and recognition enhancements [127]. Another regulator is bFGF (basic fibroblast growth factor), which if injected into the hippocampus, could prevent neuronal damage and improve memory problems associated with AD in rats [134]. An innovative biodegradable nose-to-brain drug-delivery system was obtained starting with *Solanum tuberosum lectin* (STL), which was conjugated to PLGA NPs (STL-NPs). STL-NPs demonstrated brain-targeting efficiencies in different brain tissues that were 1.89–2.45 times higher than unmodified NPs [135]. 

In further experiments, bFGF was incorporated into PEG-PLGA NPs and combined with *Solanum tuberosum lectin.* Following the administration, these NPs significantly improved spatial learning and memory in rats used as AD animal models [130]. The achieved improvements are explained thanks to the reported increased amount of delivered NPs in the brain region than those administered through the intravenous route.

Amyloid β inhibitors/modulators were also used as specific ligands; biodegradable PLGA NPs with trimethylated chitosan were synthesized without surface functionalization [136]. Brain uptake studies showed higher storage of the modified PLGA NPs in several brain districts, such as the cortex, choroid plexus epithelium, and the third ventricle regions compared with naked PLGA particles [130].

Several in vitro, in vivo, and ex vivo assays were implemented in order to evaluate the ability of curcumin and its derivatives to bind to amyloid accumulations [130].

In particular, some in vitro tests showed that a natural compound such as curcumin was a suitable tool to disrupt the amyloid peptide aggregates and disassemble the produced amyloid peptide fibrils [130]. Indeed, curcumin possesses antitau activity by inhibiting tau hyperphosphorylation and it is also involved in disrupting the tau protein tangle [130]. Significantly reduced brain Aβ levels and decreased astrocytic marker, glial fibrillary protein (GFAP), were observed after curcumin administration [130]. In addition, many specific properties are associated with this compound, for example, it promotes neuroplasticity, inhibits the activity of the acetylcholinesterase [130], and exhibits a neuroprotective effect in AD patients. This is due to the inhibitive role induced by free radicals in Aβ aggregation, inflammatory pathways, and degeneration. In vivo studies performed with PLGA NPs containing curcumin (C-NPs) showed that both free curcumin and C-NPs crossed the BBB but using only C-NPs, the curcumin mean residential time (MRT) in the brain significantly increased compared with the free compound [130]. So, this nanoformulation effectively induced neural stem cell proliferation and neuronal cell differentiation. In addition, the gene expression of nestin, reelin, Pax6, neuregulin, neurogenin, Stat3, and neuroD1, which were involved in the specific pathways, were enhanced [130]. Also, the results of in vivo studies that administered C-NPs in Alzheimer-induced Aß rat models demonstrated reversed learning as well as memory defects [130].

Quercetin exhibits neuroprotective effects against oxidative-stress-induced neurotoxicity; the related protective role of Aβ [130] was evaluated in cultures of primary rat hippocampal neurons. PLGA NPs functionalized with quercetin have inhibited and disassembled Aβ 42 fibrils. In behavioral studies, ameliorated cognition and memory deficits in APP/PS1 mice were observed by administering PLGA NPs of quercetin, and the in vivo toxicity assessment did not highlight any organ damage [137]. These animal models are double transgenic mice expressing a chimeric mouse/human amyloid precursor protein and a mutant human presenilin-1, both directed at CNS neurons.

*Ginseng* contains many ginsenosides that are known to inhibit the release of pro-inflammatory mediators [130], making them useful for treating AD. In particular, ginsenoside Rg1 reduced cerebral ischemia through the downregulation of PAR-1 expression [138]. It avoided BBB disruption in a brain injury rat model by downregulating the expression of aquaporin 4 [139]. PLGA NPs of ginsenoside Rg3 and thioflavin T (Aβ diagnostic agent) were prepared and studied in a BBB model to test their ability to cross this selective barrier. Angiopep-2, a cell-penetrating peptide, combined with PLGA NPs of ginsenoside Rg3, enhanced delivery across the BBB compared with the same NPs in the absence of Angiopep-2 [140]. 

*Bacopa monnieri*, well-known as *Brahmi*, is a water plant that contains two saponins, named bacoside-A and bacoside-B; in particular, bacoside-A considerably enhances memory acquisition, consolidation, and memory retention [130]. The neuroprotective role of *Brahmi* plant extract was studied against both Aβ (25–35) protein and glutamate-induced neurotoxicity by evaluating its effects on primary cortical culture neurons. As a result of these experiments, the extract showed a neuroprotective effect against Aβ-induced cell death but failed in its protective action against glutamate-induced excitotoxicity [141]. PLGA NPs of bacoside-A, one of the types of saponins present in the plant extract, coated with polysorbate-80 were administered to Wistar albino rats to evaluate their brain-targeting abilities. A higher bacoside-A concentration in the brain compared to the free bacoside-A solution was detected using PS-80-coated PLGA NPs [142].

Nanomedicine approaches are focusing on ROS-mediated mechanisms involved in the pathogenesis of NDs. It was observed that a relevant portion of the neuron loss in ALS is potentially due to damage caused by ROS as a result of dysfunctional Superoxide dismutase (SOD) [37]. Physiological levels of SOD1 related to motor neurons can be restored using PLGA-based NPs containing SOD1. These nanostructures transported the enzyme to the neuronal cells, protecting them against hydrogen peroxide, which triggers oxidative stress in vitro [143].

PLGA NPs containing donepezil, an acetylcholinesterase inhibitor, were coated with polysorbate-80 and radiolabeled before their intravenous administration in rats. By comparing the nanoformulation with the free drug solution, it was found that the formulated NPs assured great delivery into the brain [144]. Subsequently, PLGA-block-PEG NPs of donepezil were prepared and then studied in order to assess their destabilizing activity on fibril formation (Aβ (1–40) and Aβ (1–42)) in vitro. After obtaining this evidence, the ability of this conjugate to pass through the BBB to reach the brain was assessed in in vitro BBB model studies [145]. The structural arrangement of the polarized layer was obtained using HBMEC (Human Brain Microvascular Endothelial cells) plated on translucent membrane inserts of a transwell system (Corning) coated with an attachment factor. In the BBB crossing experiment, 1 lg/mL of free donepezil and donepezil-loaded NPs containing 1 lg/mL of donepezil, were added at different concentrations for a comparative analysis at different time-points [145]. 

Kou and Tsai achieved a drug-delivery system based on polyacrylamide (PAAM)-cardiolipin (CL)-PLGA NPs functionalized by 83–14 monoclonal antibodies (MAb) to deliver rosmarinic acid and curcumin. After crossing the BBB, this system was shown to enhance the viability of SK-N-MC cells (from a human neuroblastoma) with β-amyloid (Aβ) deposits [146].

#### 6.2.3. Chitosan Nanoparticles (CS NPs)

Chitosan is a cationic, biocompatible, and biodegradable polymer that derives from the chitin of crustacean shells and also from the cell walls of fungi [147]. Powerful formulations were prepared by modifying chitosan NPs. For instance, some of them were prepared by the ionic gelation method and loaded with galantamine, a reversible acetylcholinesterase inhibitor. This nanoformulation has extended the galantamine release (58.07% ± 6.67 after 72 h), improving formulation stability at 4 °C in terms of drug leakage and particle size, and maintaining the unaffected physicochemical properties of the free CS-NPs (*p* > 0.05) [148]. Through rhodamine labeling, these galantamine-loaded CS-NPs were detected in the olfactory bulb, hippocampus, and orbitofrontal and parietal cortexes, thus reporting successful drug delivery after IN administration [148]. The same synthetic procedure was adopted to obtain Estradiol-loaded CS-NPs, effectively improving nasal absorption and targeting the brain in rats [149]. It is known that 17β-Estradiol, a steroid female sex hormone, influences sexual characteristics and at the same time plays a relevant role in the regulation of brain development. In this regard, long-term estrogen replacement emerged as a beneficial strategy for the prevention and treatment of Alzheimer’s disease [150]. After IN administration of Estradiol-loaded CS-NPs, hormone levels were significantly higher in the CSF compared to IV administration [149].

In another study, CS-NPs loaded with pramipexole dihydrochloride (P-CS NPs), a drug currently used to treat Parkinson’s symptoms, enhanced the antioxidant status by increasing the enzymatic activities of SOD and catalase in addition to elevating dopamine levels in the brain of male Sprague–Dawley rats [151]. From these in vivo pharmacodynamic studies, the comparative findings of behavioral testing revealed increased locomotor activity and reported catalepsy in the P-CS NP treatment group compared to its nasal solution or oral-marketed tablets [151].

Piperine is a compound that significantly reverted memory impairment and neurodegeneration in the hippocampal region in the AD animal model [130]. Starting with this, the behavioral studies performed on male Wistar rats showed a significant improvement in cognitive function promoted by Piperine-based NPs. Furthermore, the neurotoxicity studies reported that the formulations were safe for the brain [152].

Also, Lycopene can be encapsulated into spherical CS-NPs with PS-80 and phosphatidylserine. These NPs improved the antioxidant enzymatic activity of catalase (CAT), superoxide dismutase (SOD), and glutathione peroxidase (GSH-PX) while delivering lycopene (5 mg/kg) through the BBB [153]. Ameliorated behavioral and cognitive impairments were also reported [153]. Ellagic acid-loaded PEG-CSNPs (EA@PCS), synthesized using a green strategy, reported a synergistic effect to prevent oxidative stress in neuronal cell lines [154].

A potential therapeutic system for AD was obtained with dextran sulfate/chitosan (DS/CS)-coated zein NPs loaded with crocin. These conjugates exhibited a better-controlled release and stronger antioxidant activity than the uncoated NPs [155]. In particular, the ELISA assay performed on the AD cell model showed a reduced concentration of amyloid β (Aβ (1–42)) in SH-SY5Y cells, ranging from 300 pg/mg in the model down to 170 pg/mg in the conjugates [155].

Other experimental results suggested the efficient silencing of the P-glycoprotein (P-gp) gene in a BBB model using siRNA-CS NPs [40]. P-gp is a multidrug-resistant protein encoded by the MDR1 gene that belongs to the family of ATP-binding cassette transporters. This protein is located at the BBB level and restricts a variety of drugs from reaching their specific therapeutic targets. Hence, a considerable reduction in P-gp substrate efflux was shown combined with the improved delivery and efficacy of doxorubicin used as a model drug [156]. In this regard, the NP-mediated delivery of anti-P-gp siRNA could represent a promising approach to selectively treating NDs.

#### 6.2.4. Carbon Nanomaterials

Carbon-based nanomaterials with hydrophobic surfaces, including zero-dimensional fullerene (C60), one-dimensional carbon nanotubes (CNTs), and two-dimensional graphene, have produced interesting results in the nanomedicine field due to their unique combinations of chemical and physical properties (i.e., thermal and electrical conductivity, high mechanical strength, and optical properties) [157].

Electroactive materials have been investigated as the next generation of neuronal tissue engineering scaffolds, which are useful after brain injury for enhancing neuronal regeneration and functional recovery [40]. In this regard, graphene is an emerging neuronal scaffold material with charge-transfer properties that has shown neuronal cell survival and differentiation in in vitro assays [158]. Chitosan and carrageenan were used as precursors dissolved in water solvent in a green way to synthesize graphene oxide sheets (GNSs) [159]. In another work, reduced graphene oxide-based hydrogels were obtained through the exfoliation method, using vitamin C solution as a chemical exfoliation solvent. In the literature, some materials of bio-origin are used as precursors for the synthesis of graphene and doped carbon nanomaterials, for example, sodium alginate, bagasse, peanut shells, grass as *Hybrid pennisetum*, clove extract, shellac flakes, and lignin and reducing sugars as glucose [159]. CNTs and graphene nanosheets (GNSs) were also produced starting with sugarcane bagasse (SCB), depending on the type of catalyst used, using the pyrolysis method [159].

Fullerenols are derivatives of hydroxyl-functionalized fullerenes with a regular arrangement of carbon atoms and exhibit antioxidant properties [160]. Their neuroprotective effect was demonstrated in both in vitro and in vivo tests; in particular, in cultured cortical neurons, their excitotoxicity and apoptotic process were limited, whereas in a familial ALS mouse model it delayed the onset of motor degeneration [37].

Carbon nanotubes are commonly used as carbon-based nanomaterials and their unique structure provides exceptional electrical, mechanical, optical, and thermal properties, as well as a high surface area [161]. They are mainly used in nanomedicine as nanocarrier systems to deliver drugs, hormones, and enzymes, but are also used in gene therapy, tissue engineering, and biosensing [161]. Single-walled CNTs with a size range of 27–31 nm, were obtained using a synthetic route adopting nickel(II) chloride (NiCl_2_) (5% wt.) as the catalyst and olive oil as the carbon precursor; such size uniformity was not observed when using coconut oil. This was due to the high content of saturated fats (82.5%) in coconut oil, which are less reactive compared to unsaturated hydrocarbons [159]. Other CNT synthetic routes start with different bio-based/waste materials as the catalyst or carbon source including walnuts and the waste biomass of *Typha orientalis* [159]. However, neurotoxic effects are also associated with this kind of nanostructure including astrogliosis, neuroinflammation, apoptosis, increased oxidative stress, and BBB destruction [162].

Extreme caution should be exercised when considering these nanomaterials for brain delivery even though a precise functionalization process is possible by modifying their physical and biological properties. The neurotoxic effects could be modulated by tuning a series of carbon nanotube parameters such as their diameters or lengths, structures, concentrations, and impurities [163]. On the other hand, it was proven that carbon nanotubes were particularly effective and widely used in the development of nanoscaffolds for neuronal growth [70]. Recently, in an experimental study, the therapeutic effects of single-walled carbon nanotubes (SWNTs) were investigated in a rat model of binge alcohol-induced neurodegeneration. Four types of SWNT structures were tested (iSWNT, bSWNT, dSWNT, and bSWNT) and particularly from the application of the bundled SWNTs (bSWNTs), learning and memory restoration were observed [164]. The peculiar ability of bSWNTs to directly interact with neurotrophic receptors, especially tropomyosin-related kinase B (TrkB) receptors such as endogenous neurotrophins, was assessed. Such novel “artificial neurotrophins” serve as active therapeutic components, triggering specific signals involved in neuroprotection and neuroregeneration [164], and are suitable against a number of human diseases such as autism, schizophrenia ALS, AD, PD, and alcohol exposure.

Fullerenes perform a protective action via two principal methods: radical sponge and hydrophobic surface [40]. The unique “radical sponge” structure of fullerene entraps several radicals in a single spherical molecule with effective antioxidant activity against the cytotoxicity induced by oxidative stress [165]. An interesting study describes the synthetic process of water-soluble C60 fullerene derivatives with different types of linkages between the fullerene cage and the solubilizing addend (compounds 1−3: C−C bonds, compounds 4−5: C−S bonds, compound 6: C−P bonds, and compounds 7−9: C−N bonds) [166]. Fullerene derivatives 1−6 were observed to induce neural stem cell (NSC) proliferation in vitro and preserve the function of injured CNS in a Zebra fish animal model. Conversely, fullerene derivatives 7−9 inhibited glioblastoma cell proliferation in vitro, reducing glioblastoma formation in Zebra fish. These effects were then correlated with changes affecting cellular metabolism. 

Then, surface functional groups affected the properties and interactions of C60 with NSCs and glioma cells, acting either as a neuroprotective or antitumor tool for any treatment of CNS-related diseases, as confirmed in a cellular model of Parkinson’s disease [166]. The neuroprotective effect was also shown in vitro by carboxy fullerene SOD mimetics, cell-permeable molecules [167] with a SOD-like activity highly reactive to superoxide radicals. A facile, mild, and green synthesis approach was proposed for water-soluble C60 NPs capable of ROS-scavenging by combining mussel-inspired chemistry with the Michael addition reaction [168]. The produced biocompatible C60-PDA-GSH NPs (consisting of polydopamine and reduced glutathione) displayed great free radical scavenging activity, thus exhibiting a cytoprotective effect against oxidative stress at a low concentration of 2 μg/mL [168]. Their antioxidant performance was assessed in four kinds of cell lines: human epidermal keratinocytes (HEK-a), human umbilical vein endothelial cells (HUVEC), human microglia (HM), and normal liver cells (L-02) cells.

#### 6.2.5. Lipid Nanocarriers

Lipid-based nanosystems are highly biocompatible and biodegradable colloidal carriers that are attractive for brain drug targeting. Their peculiar low cytotoxicity is due to the use of physiological lipids, and they are generally recognized as safe excipients [73]. Both lipophilic and hydrophilic drugs for brain delivery could be encapsulated in this kind of system, revealing high entrapment, and loading efficiency for hydrophobic drugs and superior control over drug release kinetics by providing long-term release [73]. Thanks to their lipophilic nature and small size, these nanocarriers show a tendency to cross the BBB, inducing a drug gradient from the blood to the neuronal tissues, with the consequent enhancement of drug bioavailability, retention time, and uptake in the brain [10]. The synthesis process is cheap and could be easily scaled up [10]. Furthermore, some of the lipid-based nanosystems (i.e., SLN) exhibit better physicochemical stability. In contrast to these benefits, it is important to underline that lipid nanocarriers are able to confine only low doses of hydrophilic drugs as well as only some types of proteins and peptides [169].

#### 6.2.6. Solid Lipid NPs (SLNs)

Solid-lipid nanoparticles (SLNs) represent the new generation of colloidal nanocarriers that are based on lipid components unlike phospholipids (as triglycerides, glyceride mixtures) that are solid both at room and body temperatures [170,171].

They act as an alternative approach to liposomes. Their structure is characterized by a hydrophobic solid matrix core suitable for better entrapment and a more efficient load of hydrophobic drugs than conventional nanocarriers [172] and they are associated with finely controlled drug release and improved stability. Typical solid lipid molecules able to produce SLNs are stearic acid, cetyl alcohol, cholesterol butyrate, carnauba wax, beeswax, and emulsifying wax [170]. SLNs are characterized by a size range of between 50 and 1000 nm, like polymeric NPs [172]. As opposed to the latter, SLNs show peculiar properties such as biocompatibility, bioavailability, drug protection, good tolerance, and biodegradability in generating safe products [171]. Furthermore, their synthetic process is cheap, with the possibility of scaling up the procedure [10]. Their high lipophilic nature allows them to naturally cross the BBB and target the CNS; therefore, SLNs have been extensively used as nanovectors for the delivery of antitumoral drugs to the brain [173,174]. The brain uptake of SLNs takes place through a paracellular pathway by opening the TJ in the brain microvasculature through passive diffusion, active transport, and endocytosis. In addition, by properly modifying the surface of SLNs with apolipoprotein E, it is possible to improve brain-targeted drug delivery due to the predominant expression of ApoE receptors in the brain region [173]. Active targeting consists of binding ligands to the surface of the NPs to promote interaction with proteins that are constitutively expressed at the BBB such as the LDL receptor transferrin (Tfr) or the insulin receptor. This kind of modification increases the selectivity of the uptake via receptor-mediated transcytosis (RMT) and in addition, protects NPs from enzymatic degradation [174].

An important aspect to be considered is opsonization, that is, the adsorption of plasma proteins, which makes the nanosystems more perceptible to phagocytic cells. This physiological process determines the fast clearance of NPs from the bloodstream in the liver and spleen through RES (reticuloendothelial system) phagocytosis [42,74]. This process could be easily decreased by conjugating specific polymers on the surface of the lipid nanocarrier that can be natural (e.g., dextran, alginate, and chitosan) or synthetic (e.g., poly(ethylene glycol)(PEG), polysorbates) hydrophilic polymers [10]. The hydrophilic external layer protects nanosystems from macrophages, providing a longer circulatory time and promoting the translocation to the brain [173]. 

Bondi et al. developed and tested riluzole-based SLNs for the treatment of ALS [175]. Riluzole belongs to the benzothiazole class and by comparing it with riluzole-based SLNs separately administered in a rat model, researchers have discovered that these functionalized lipid nanosystems were able to bypass the BBB [175]. Additionally, an increased amount of the drug was delivered to the brain compared to the free riluzole. Several studies have also reported a decrease in side effects by using riluzole-based NPs instead of conventional drugs, at the organ level (e.g., heart, liver and spleen, kidneys) due to the lower amounts of the loaded compound [175]. Another case in which drug-based NPs have obtained better results compared to the free drug was reported with Galantamine SLNs. After oral administration in cognitive deficit rats, these NP formulations significantly restored memory, also enhancing bioavailability by about 100% [176]. Memory recovery in isoproterenol-induced cognitive deficit rats after the administration of galantamine-loaded SLNs, confirmed the efficacy of the nanoformulation to deliver more galantamine into the brain [176] compared with the free drug. Green tea contains polyphenolic compounds such as epigallocatechin gallate, (−)-epigallocatechin, (−)-epicatechin, and (−)-epicatechin-3-gallate (EGCG), which are able to interfere in different pathways associated with AD pathogenesis, in particular in the reduced production of Aβ [130]. EGCG showed modulation of apoptosis and APP processing [177]. Nanolipid particles based on EPCG significantly improved neuronal α-secretase in in vitro assays on SweAPP N2a cells, a family of proteolytic enzymes that cleave amyloid precursor protein (APP) in its transmembrane region, and increased oral bioavailability was also reported in animal models [178]. In order to assess the efficacy of entrapped nutraceutical compounds, Bhatt et al. explored the potential of rosmarinic acid for the control of HD [45]. Rosmarinic-acid-loaded SLNs coated with polysorbate 80 and with a mean size of 149.2 ± 3.2 nm, were successfully developed for IN delivery and tested on an HD rat model. The drug concentration was maintained by the proposed formulation for up to 14 h, thus allowing a potential reduction in the dosing frequency and enhancing the effectiveness of the therapy. As a result, SLNs significantly attenuated the induced behavioral abnormalities, as well as deficits in locomotor activity and body weight [45]. Also, an important decrease in striatal oxidative was reported compared to the same NPs injected IV or to the free rosmarinic acid [45]. Moreover, from the perspective of AD, quercetin was encapsulated into SLNs with a size of around 200 nm and functionalized with transferrin [179]. Cytotoxicity assays were performed on the hCMEC/D3 cell line, which was characterized by a brain endothelial phenotype. Even at the highest concentration (30 μM), these NPs did not cause evident toxic effects after 4 h of exposition and better BBB permeation was revealed [179]. In another work, researchers formulated astaxanthin-SLNs and then tested them in a PC12 cell line, in which the oxidative stress phenomena was given by H_2_O_2_. The reported neuroprotective effect could provide a useful guide for treating NDs [180].

A summary of the above-mentioned NPs was reported in Table 1.

## 7. Challenges and Limitations

The widespread application of nanotechnology in the biomedical field has raised some concerns regarding its potential health risks as opposed to the associated benefits. The numerous physicochemical properties of nanomaterials might cause adverse effects in living organisms [181] under particular conditions. The properties of tunability could determine unpredictable biological responses when introduced in vivo [181], and the high reactivity, as well as the colloidal instability, allow for nanomaterial aggregation [182]. Moreover, several studies reported the precise and efficient conveyance of nanomaterials from the administration site to the secondary vital organs including the brain, liver, heart, lungs, and kidney [37]. In vivo experiments allow for the evaluation of the potentially toxic effects at the accumulation site by analyzing organ toxicity and carcinogenicity [183]. The administration of nanomaterials for neurological treatments could activate neurotoxicity. These adverse effects could manifest immediately or after a period of time and could have reversible or permanent implications for parts of the nervous system or the whole system [162]. Ideally, after a controlled release of the encapsulated therapeutic drug molecules, the residual NPs should be safely degraded and excreted from the body [33].

The appearance of a biocorona on the surface of nanomaterials represents a phenomenon that occurs in biological systems. This biocorona could affect the NPs’ physicochemical properties, functionality, and biodistribution, inducing highly toxic effects [184]. Another important point is the limited number of standardized model systems, experimental assays, and in vivo monitoring systems for accurately determining the toxic effects of nanostructures. The BBB is a highly dynamic barrier whose properties change depending on physiological or pathological conditions. Available BBB models require the use of (i) primary co-cultures of mouse brain endothelial cells and astrocytes, (ii) primary mono-, co-, and triple-cultures of rat endothelial cells/astrocytes/pericytes, (iii) bovine co-cultures of endothelial cells and astrocytes, (iv) porcine monocultures of endothelial cells, and (v) human cultures using either the cCMEC/D3 endothelial cell line or stem cells [73]. Therefore, such cellular models need to be refined to allow for the translation of the results into in vivo contexts. In order to overcome this limitation, the implementation of lab-on-a-chip techniques and organ cultures represents efficient and accurate strategies.

## Figures and Tables

**Table 1:** Beneficial effects of different kinds of NPs in in vitro and in vivo models.

NPs and NMscomposition	Green Extract	Functionalization and Encapsulation	Beneficial Effect	In Vitro/In Vivo Model	References
Cerium oxide (CeO_2_) NPs	-	-	Antioxidant properties	SH-SY5Y	[75]
Selenium (Se) NPs	-	Resveratrol (Res)	Antioxidant and antiaggregatory properties	PC12 cells	[80]
	-	Chondroitin sulfate (ChS)	Protection from Aβ (1–42)-induced cytotoxicity; reduced level of ROS, malondialdehyde (MDA), and hyperphosphorylation of tau	SH-SY5Y	[80]
	-	Glycine	Neuroprotection, antioxidant role decreasing MDA levels, and regulating SOD, GSH-PX enzymes	PD-rats	[80]
Gold (Au) NPs	*Hypericum hookerianum*	-	Antiparkinson-like effect	Swiss albino mice	[86]
	*Paeonia moutan*	-	Alleviated neuroinflammation and improved motor coordination	Murine microglial BV2 cells and PD-induced C57BL/6 mice	[87]
	*Cinnamomum verum*	-	Depletion of induced oxidative stress and motor abnormalities	PD-rats	[90]
	*Ephedra Sinica*	-	Depletion intopro-neuroinflammatory cytokines and mediators; reduced ROS levels	Mouse primary microglia and immortal BV-2 mouse microglial cells	[91]
	-	Anthocyanin	Ameliorated memory impairments; protective role in pre- and post-synapticproteins	Aβ (1–42) mouse	[92]
	-	Engineered β-sheet breaker peptide (CLPFFD)	Increased permeability in the brain; Disrupted Aβ toxic aggregates	Co-cultured bovine microvessel brain endothelial cells and newbornratastrocytes; Male Sprague–Dawley rats	[94]
Silver (Ag) NPs			Regulation of gene and protein expressions of Aβ depositions	Rat brain microvessel vascular endothelial cells (BMVECs)	
	*Lampranthus coccineus* and *Malephora lutea*	-	Anti-Alzheimer and antioxidant activity	AD-inducedrats	[104]
	*Melia azedarach*	-	increased antioxidant activity		[105]
	*Erythrina* *suberosa*	-	ROS scavenger	A-431 osteosarcoma cell line	[106]
	*Pulicaria undulata* L.	-	Prevented amyloid aggregation	α-lactalbumin (amyloid model)	[107]
PEG-coated Fe_3_O_4_ NPs	-	Lactoferrin	Enhanced permeability across the BBB	Primary porcine and bovine brain capillary endothelial cells (PBCECs); Sprague–Dawley rats	[115]
Dextran-coated Fe_3_O_4_ NPs	-	Quercetin	Enhanced bioavailability	Wistar male rats	[118]
Fe_3_O_4_ NPs	-	W20 antibody and XD4 peptide	Microglialphagocytosis of AβO ligomers, restoration of cognitive deficits, and alleviated neuropathology of AD	SH-SY5Y cells; AD mice	[119]
PVP-SPIONs	-	1,2-Dimyristoyl-sn-glycero-3-phosphocholine (DMPC)	Fast brain delivery, activation of neuron membrane channels	Rat adrenal pheochromocytoma (PC-12) cells; Sprague–Dawley rats	[120]
	-		Great brain uptake, decrease in brain Aβ deposition	APP2576 transgenic mice	
PEG-PLGA NPs	-	Lactoferrin	Optimal drug delivery to the brain	16HBE and SH-SY5Y cells	[132]
	-	Lactoferrin and Coumarin-6	Brain parenchyma-targeting ability, high cellular uptake,	Mouse brain endothelial cell line (b.End3); BALB/c mice	[133]
PS80-PBCA NPs	-	Nerve growth factor (NGF)	Reversed scopolamine-induced amnesia, improved memory and recognition, reduction of the basic symptoms of Parkinsonism	C57Bl/6 mice	[127]
PEG-PLGA NPs	-	*Solanum Tuberosum Lectin* (STL)	High brain-targeting efficiency; noninvasive brain drug-delivery system	Calu-3 cells (human lung adenocarcinoma); Sprague–Dawley rats	[135]
	-	STL and basic fibroblast growth factor (bFGF)	Neuroprotective effect, improved spatial learning and memory	Sprague–Dawley rats	[130]
PLGA NPs					
	-	Curcumin	Neural stem cell proliferation and neuronal cell differentiation; reversed learning and memory defects	AD-induced rats	[130]
	-	Quercetin	Inhibited and disassembled Aβ 42 fibrils; ameliorated cognition and memory deficits	SH-SY5Y cells; APP/PS1 mice	[137]
	-	Angiopep-2, Thioflavin T, ginsenoside Rg3	Reduction of Aβ plaques, decreased ROS generation, inhibiting Aβ-mediated neuronal mitochondrial stress	C6 ratglial cells and THP-1 human monocytic cells	[140]
PS80-PLGA NPs	-	Bacoside-A	Brain targeting nanodelivery, sustained release pattern	Wistar albino rats	[142]
PAAM-CL-PLGA NPs	-	83–14 MAb, rosmarinic acid, curcumin	Enhanced viability in the presence of β-amyloid (Aβ) deposits	SK-N-MC cells (human neuroblastoma)	[146]
Chitosan (CS) NPs	-	Estradiol	Improved nasal absorption and brain targeting	Wistar rats	[149]
	-	Pramipexole dihydrochloride	Antioxidant role; enhancement of dopamine level in the brain, increased locomotor activity	Sprague–Dawley rats	[151]
	-	Piperine	Improvement in cognitive function	Wistar rats	[152]
PS80-CS NPs	-	Lycopene, Phosphatidylserine	Improved antioxidant enzymatic activity of CAT, SOD, GPx; Ameliorated behavioral and cognitive impairments	Albino mice	[153]
PEG-CS NPs	-	Ellagic acid	Prevent oxidative stress in vitro	SH-SY5Y cells	[154]
Fullerenols and fullerene	-	-	Neuroprotective effect, limited excitotoxicity and apoptosis; delayed onset of motor degeneration	Cortical neurons; familial ALS mouse model	[37]
Single-walled carbon nanotubes (SWNTs)	-	-	Learning and memory restoring	Sprague–Dawley rats	[164]
Fullerene derivatives	-	-	Induced proliferation of NSC; preserved CNS functions	Neural stem cell (NSC); Zebra fish	[166]
Carboxy fullerene	-	SOD mimetics	Neuroprotection	Cortical neurons	[167]
C60 NPs		Polydopamine (PD) and Reduced Glutathione (GSH)	Free radicals scavenging	HEK-a, HUVEC, HM, L02 cell lines	[168]
Nanolipid particles	-	Epigallocatechin-3-gallate (EGCG)	Improved neuronal α- secretase	SweAPP N2a cells	[178]
PS-80 SLN	-	Rosmarinic acid	Attenuated behavioral, locomotor, and body weight deficits	HD rat model	[45]
SLN	-	Quercetin and transferrin	BBB permeation	hCMEC/D3 cell line	[179]
	-	Astaxanthin	Neuroprotection	PC12 cell line	[180]

## Data Availability

Not applicable.
